# Using QALYs as an Outcome for Assessing Global Prediction Accuracy in Diabetes Simulation Models

**DOI:** 10.1177/0272989X241285866

**Published:** 2024-10-30

**Authors:** Helen A. Dakin, Ni Gao, José Leal, Rury R. Holman, An Tran-Duy, Philip Clarke

**Affiliations:** Health Economics Research Centre, Nuffield Department of Population Health, University of Oxford, UK; Health Economics Research Centre, Nuffield Department of Population Health, University of Oxford, UK; Centre for Health Economics, University of York, York, UK; Health Economics Research Centre, Nuffield Department of Population Health, University of Oxford, UK; Diabetes Trials Unit, Radcliffe Department of Medicine, University of Oxford, UK; Centre for Health Policy, Melbourne School of Population and Global Health, University of Melbourne, Australia; Health Economics Research Centre, Nuffield Department of Population Health, University of Oxford, UK

**Keywords:** type 2 diabetes mellitus, quality-adjusted life-years, patient-level simulation, risk modeling, model performance, microsimulation

## Abstract

**Objectives:**

(1) To demonstrate the use of quality-adjusted life-years (QALYs) as an outcome measure for comparing performance between simulation models and identifying the most accurate model for economic evaluation and health technology assessment. QALYs relate directly to decision making and combine mortality and diverse clinical events into a single measure using evidence-based weights that reflect population preferences. (2) To explore the usefulness of Q^2^, the proportional reduction in error, as a model performance metric and compare it with other metrics: mean squared error (MSE), mean absolute error, bias (mean residual), and *R*^2^.

**Methods:**

We simulated all EXSCEL trial participants (*N* = 14,729) using the UK Prospective Diabetes Study Outcomes Model software versions 1 (UKPDS-OM1) and 2 (UKPDS-OM2). The EXSCEL trial compared once-weekly exenatide with placebo (median 3.2-y follow-up). Default UKPDS-OM2 utilities were used to estimate undiscounted QALYs over the trial period based on the observed events and survival. These were compared with the QALYs predicted by UKPDS-OM1/2 for the same period.

**Results:**

UKPDS-OM2 predicted patients’ QALYs more accurately than UKPDS-OM1 did (MSE: 0.210 v. 0.253; Q^2^: 0.822 v. 0.786). UKPDS-OM2 underestimated QALYs by an average of 0.127 versus 0.150 for UKPDS-OM1. UKPDS-OM2 predictions were more accurate for mortality, myocardial infarction, and stroke, whereas UKPDS-OM1 better predicted blindness and heart disease. Q^2^ facilitated comparisons between subgroups and (unlike *R*^2^) was lower for biased predictors.

**Conclusions:**

Q^2^ for QALYs was useful for comparing global prediction accuracy (across all clinical events) of diabetes models. It could be used for model registries, choosing between simulation models for economic evaluation and evaluating the impact of recalibration. Similar methods could be used in other disease areas.

**Highlights:**

- Diabetes simulation models are currently validated by examining their ability to predict the incidence of individual events (e.g., myocardial infarction, stroke, amputation) or composite events (e.g., first major adverse cardiovascular event).
- We introduce Q^2^, the proportional reduction in error, as a measure that may be useful for evaluating and comparing the prediction accuracy of econometric or simulation models.
- We propose using the Q^2^ or mean squared error for QALYs as global measures of model prediction accuracy when comparing diabetes models’ performance for health technology assessment; these can be used to select the most accurate simulation model for economic evaluation and to evaluate the impact of model recalibration in diabetes or other conditions.

More than 20 cost-effectiveness models of type 2 diabetes have been developed,^1^ most of which use microsimulation and simulate one patient at a time.^2^ Many use an integrated set of risk equations predicting mortality and clinical events (e.g., myocardial infarction [MI], stroke, or amputation) that were estimated on individual-patient data from the UK Prospective Diabetes Study (UKPDS) trial.^3,4^

A key criterion for assessing the accuracy with which diabetes simulation models predict individuals’ outcomes is external validity (i.e., the degree to which they can replicate the incidence of events in samples not used to build the model).^5^ At least 5 studies have validated the UKPDS Outcomes Model version 2 (UKPDS-OM2),^4,6–9^ all of which compared observed and predicted cumulative incidence of individual clinical events. Diabetes models are typically able to predict the incidence of many clinical events (e.g., MI, stroke, blindness).

However, external validity may vary between health outcomes and/or between performance metrics. External validation studies need to assess the outcome measure that is most relevant for the intended application. Focusing on specific events may be insufficient when validating a model for health technology assessment (HTA), where we are interested primarily in the accurate prediction of life expectancy and of quality-adjusted life-years (QALYs).^10^ Interactions between events^4^ also mean that recalibrating one equation may affect the incidence of other events, necessitating an outcome capturing all events.

For external validation and calibration studies, prespecifying a single outcome and a single performance metric in an analysis plan can minimize reporting bias^11^ and make it easier to choose between large numbers of recalibrated models. However, using multiple measures can provide a more nuanced comparison of the strengths and weaknesses of the models under comparison.

A global accuracy measure capturing all relevant outcomes would be a useful addition to existing methods to inform evaluations of individual model performance and comparisons between simulation models. Such a measure could be used to choose a model for economic evaluation or HTA, inform model registries (e.g., Mount Hood^1^), and evaluate the impact of model recalibration.

Previous validation studies have evaluated prediction accuracy for commonly used trial composite outcomes (e.g., time to first atherosclerotic event^12^). These may be useful for external validation to inform trial design or extrapolation of clinical endpoints. However, they may be less relevant to HTA and give equal weight to all events within the composite (e.g., MI, stroke, cardiovascular death) but no weight to recurrent events or events not included in the composite outcome.

In this article, we propose using QALYs as a global measure of health to facilitate more accurate and generic comparisons of the prediction accuracy of diabetes simulation models used for economic evaluation or HTA. To our knowledge, such an approach has not been explored previously. QALYs combine data on mortality and nonfatal clinical events that reduce patients’ health-related quality of life. The weights attached to different clinical events are based on health state preference values estimated using choice-based methods, such as time tradeoff. Following the reference case of many HTA organizations,^13,14^ these weights are based on general population preferences.^15^ Evaluating model accuracy using QALYs reflects the way that models are used for HTA^10^ and combines diverse events/dimensions into a single measure on which model performance can be ranked.

External validation and choosing between models also require selection of a primary metric of prediction accuracy. Previous studies validating UKPDS-OM2^4,6–9^ followed guidelines for prognostic models when measuring prediction accuracy,^16,17^ presenting C-statistics, mean absolute percentage error, and graphical comparisons of the cumulative incidence of events. Continuous outcomes, such as QALYs, can also be evaluated using mean squared error (MSE). Q^2^ ( $1-\mathrm{MSE}/SD^{2}$ , where SD is the standard deviation across observed values) has been used in other fields to identify outliers or as a test criterion for prognostic relevance^18,19^ but to our knowledge has not previously been used in health economics.

In this article, we aim to provide a quantitative example of how QALYs could be used as a global outcome measure when comparing diabetes models. We also illustrate the benefits of Q^2^ as a metric of prediction accuracy and model performance. As an exemplar, we compared prediction accuracy between UKPDS Outcomes Model version 1 (UKPDS-OM1)^3,20^ and version 2 (UKPDS-OM2).^4,21^ Although UKPDS-OM1 and OM2 have been compared in UKPDS data and their risk equations have been compared within other models, we are not aware of any previous study directly comparing the prediction accuracy of these 2 simulation models in an external dataset. We also discuss the implications of using QALYs for this approach and describe methods/code that can be used in future studies.

## Methods

### UKPDS Outcome Models

UKPDS-OM1^3,20^ and UKPDS-OM2^4,21^ comprise individual-patient simulation models that predict clinical events and mortality for individuals with type 2 diabetes based on their clinical event history and risk factor levels at baseline and in subsequent years. Lifetime costs, QALYs, and event rates are predicted for each person in the population.

UKPDS-OM1 used data collected from 1977 to 1997 from 3,642 UK patients with newly diagnosed type 2 diabetes who participated in the UKPDS randomized trial.^3,22^ UKPDS-OM1 predicts the incidence of mortality and 7 clinically adjudicated^23^ clinical outcomes (ischemic heart disease [IHD], MI, stroke, congestive heart failure, blindness, amputation, and renal failure) based on history of events and 10 risk factors (age, ethnicity, sex, body mass index, glycated hemoglobin, lipids, blood pressure, smoking, peripheral vascular disease, and atrial fibrillation). We used the version 1.3 stand-alone software implementation.^20^

UKPDS-OM2 used data on all 5,102 UKPDS trial participants and included up to 10 additional years’ posttrial follow-up for outcomes and 5 additional years of clinical risk factor data.^4^ UKPDS-OM2 predicts 1 additional clinical outcome (diabetic foot ulcer) as well as second occurrences of MI, stroke, and amputation. Version 2.2 was used in all simulations.^21^

### External Validation Data

We externally validated UKPDS-OM1 and UKPDS-OM2 using data from the Exenatide Study of Cardiovascular Event Lowering (EXSCEL ClinicalTrials.gov NCT01144338) multinational cardiovascular outcome trial.^24^ EXSCEL evaluated the addition of once-weekly exenatide to usual care, following 14,752 participants with type 2 diabetes, with or without previous cardiovascular disease, for a median of 3.2 y between 2010 and 2017. We pooled data from intervention and control arms. After excluding 23 participants with insufficient data, 14,729 participants were analyzed. Appendix 1 describes their baseline characteristics, imputation of missing data, and data-cleaning methods.

### Outline of Analytical Methods

We simulated events for EXSCEL participants over the trial period using UKPDS-OM1 and UKPDS-OM2 using observed risk factor values as predictors. Postbaseline risk factor values were used (when available) as we primarily aimed to validate the model risk equations in the context of the model, rather than time path equations. Missing risk factor values were imputed using multiple imputation and time path equations^25,26^ (Appendix 1). We assessed how accurately the QALYs estimated by the models for each participant (“model QALYs”) predict the QALYs that this participant would have experienced if the disutility values used in the model were applied to the observed clinical events in the trial (“trial QALYs”). The level of agreement between model QALYs and trial QALYs for each participant is proposed as a measure of the accuracy of model predictions.

Model QALYs were estimated by UKPDS-OM1^3^ and UKPDS-OM2.^4^ Trial QALYs were estimated using the code shown in Appendix 2, which mirrors the assumptions used in UKPDS-OM2 to calculate QALYs (described in Appendix 3). Both model and trial QALYs indicate the total QALYs over the period for which that participant was in the trial.

The base-case analysis used the utility inputs^27^ that are defaults within UKPDS-OM2 (Appendix 3) since they were estimated on longitudinal data (UKPDS) using fixed-effects models that avoid bias from omitted time-invariant variables.^27^ Like most previous validation studies,^9,28^ our analysis was based on point estimates from the model; for simplicity, uncertainty around model predictions and utilities was not quantified. No discounting was applied to simplify the analysis and to give equal weight to all person-years of data. A sensitivity analysis explored the impact of discounting.

### Estimation of Model QALYs

**Figure 1:**
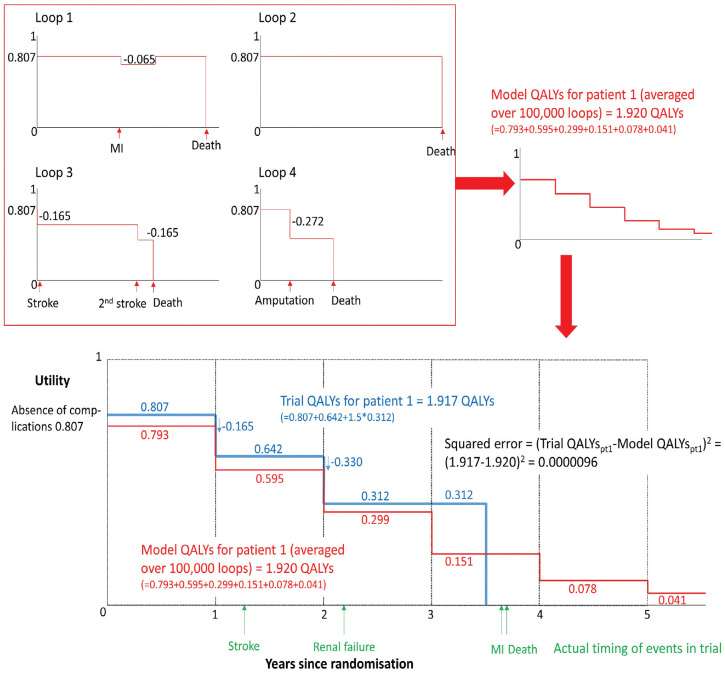
Methods and assumptions for estimating model quality-adjusted life-years (QALYs) and trial QALYs for a hypothetical trial participant. When estimating trial QALYs and estimating model QALYs for each loop of the model, all participants begin with a utility of 0.807,^27^ which may be decreased following events, based on the assumptions within UKPDS-OM2 that are described in Appendix 3. For example, in loop 1 for this hypothetical patient, myocardial infarction reduces utility by 0.065 for 1 y, while in loop 3, successive strokes permanently reduce utility by 0.165 following each event and the patient dies before the end of trial follow-up. Model QALYs for this patient are averaged over at least 100,000 loops (giving equal weight to each loop). In a proportion of loops (e.g., loop 3), the model predicts that this patient will have events or die during year 1, so the model QALYs in year 1 are 0.793 (cf. the initial utility of 0.807). Conversely, in some loops, the model predicts that the patient will survive for >4 y, so the model QALYs extend beyond the date of the patient’s death. During the trial, this hypothetical participant actually experienced a stroke in year 2, which (based on the utilities within the model) reduced utility by 0.165. Subsequently, the patient had renal failure, which reduced utility by a further 0.330, and the participant died during year 4. When estimating QALYs (whether for the trial or the model), we assumed that events occurred at the start of each year and death occurred halfway through the year. Appendix 3 describes the assumptions and utilities used.

For the base-case analysis, we ran 100,000 Monte Carlo replications (or “loops”) for each participant in UKPDS-OM1 and 1,000,000 for UKPDS-OM2 to minimize stochastic (first-order) uncertainty (i.e., random variability between patients with identical characteristics due to chance outcomes^29^), although 50,000 loops were sufficient for convergence (Appendix 5, Figure A5.3). Fewer loops were used for UKPDS-OM1 due to the longer simulation time. In each loop of the model, each participant may experience different events and/or die at different times. Each clinical event reduces participants’ utility by a certain “disutility” in the year of the event and a potentially different amount in subsequent years (Figure 1). Life-years, “model QALYs,” and event incidence were averaged across loops to obtain model predictions for each participant.

The model predicts QALYs over 7 y for all participants, regardless of whether the participant died or was censored in the trial. For each participant, “model QALYs” and model predictions of the incidence of clinical events were summed over the time until the participant was censored due to withdrawal or completion of the study. For participants who died during the trial, the censoring date for model QALYs was set to January 7, 2017 (the mean study end date for participants who did not withdraw or die before study completion) to reflect the follow-up duration the participant would have if they had survived. We adjusted model QALYs in the year in which patients were censored to allow for deaths occurring that year (Appendices 2–3).

### Estimation of Trial QALYs

We calculated “trial QALYs” for each participant by applying the default disutilities for each event from UKPDS-OM2 (Figure 1, Appendices 2–3). Although EXSCEL participants completed EQ-5D, these data were not used to calculate trial QALYs because this study aimed to assess the validity of the risk equations, not the utility inputs. Like model QALYs, trial QALYs were calculated for discrete years and assumed that events occurred at the beginning of each year and deaths occurred halfway through each year. For each participant, we added up the trial QALYs accrued in each year until the participant died or was censored. Trial QALYs during the year in which the participant died were estimated by multiplying the participant’s utilities during that year by 0.5 (reflecting the half-cycle correction within the model). Trial QALYs during the year in which the participant was censored were multiplied by the proportion of the year for which the participant remained in the trial.

### Performance Metrics

Five performance metrics were used to evaluate how accurately “model QALYs” from UKPDS-OM1 and UKPDS-OM2 predict “trial QALYs.” All metrics were based on the difference between the model QALYs for patient i (averaged over microsimulation loops) and the trial QALYs for patient i .

#### Bias

Bias is defined as the tendency for predicted values to shift in one direction from the observed values. A biased model that systematically under-/overestimates observed values would be considered unreliable^30^ or poorly calibrated.^31^ We estimated bias by averaging deviations (residuals) between model and trial QALYs across participants.

(1) $$\mathrm{bias}=\frac{\sum_{i=1}^{N}\left(M_{i}-T_{i}\right)}{N}$$

where $M_{i}$ is the mean estimate of “model QALYs” for participant i averaged over 100,000 loops, $T_{i}$ is the “trial QALYs” for participant i , and *N* is the number of trial participants. A bias value of zero indicates no bias, positive (negative) values indicate that the model overestimates (underestimates) trial QALYs, and larger absolute values indicate higher levels of bias.

#### MSE

MSE was our primary performance metric since it is increased by both bias and poor discrimination and therefore provides a good global measure of model performance. Discrimination comprises a model’s ability to predict which patients have high values and which have low values, that is, how well it captures heterogeneity and predicts high (or low) model QALYs for those participants with high (or low) trial QALYs. As the difference between observed and predicted values is squared, large differences between observed and predicted values increase MSE more than mean absolute error (MAE).

(2) $$\mathrm{MSE}=\frac{\sum_{i=1}^{N}{\left(M_{i}-T_{i}\right)}^{2}}{N}$$

#### MAE

MAE comprises the average of absolute differences between observed and predicted outcomes and is thus less sensitive than MSE to outliers or predictions that are far from the observed values, but it is also increased by both bias and poor discrimination.

(3) $$\mathrm{MAE}=\frac{\sum_{i=1}^{N}|M_{i}-T_{i}|}{N}$$

#### *R* ^2^

The coefficient of determination (*R*^2^) indicates the proportion of variability explained by a linear regression model and can be compared between samples. We estimated *R*^2^ by regressing trial QALYs on model QALYs using ordinary least squares. This regression effectively recalibrates the slope (*a*) and intercept (*b*), which means that *R*^2^ measures discrimination but does not capture bias within predictions.

(4) $$R^{2}=1-\frac{\sum_{i=1}^{N}{\left(aM_{i}+b-T_{i}\right)}^{2}}{\sum_{i=1}^{N}{\left(T_{i}-\bar{T}\right)}^{2}}$$

where $\bar{T}$ represents mean trial QALYs.

#### Q^2^

Q^2^ is a measure of the proportional reduction in error^18,19^ that captures bias and discrimination. A model perfectly predicting outcomes would have a Q^2^ of 1; unlike *R*^2^, Q^2^ can be negative for very poor models. It is analogous to scaled Brier scores^30^ but uses a different scaling formula. The absolute value for Q^2^ can be interpreted in isolation and can be directly compared between studies, subgroups, or outcome measures, unlike MSE and MAE (which tend to be larger in samples or outcomes with larger standard deviations).

(5) $$Q^{2}=1-\frac{\mathrm{MSE}}{SD^{2}}=1-\frac{\sum_{i=1}^{N}{\left(M_{i}-T_{i}\right)}^{2}/N}{\sum_{i=1}^{N}{\left(T_{i}-\bar{T}\right)}^{2}/(N-1)}$$

### Additional Analyses

We present prediction accuracy for life-years and conducted 8 sensitivity analyses to assess the robustness of the results to changes in the assumptions:

1. including second MI, stroke, amputation, blindness, and ulcer when estimating trial QALYs, regardless of patient history;
2. excluding disutilities from second events (MI, stroke, or amputation);
3. excluding disutilities from second events or ulcers;
4. using alternative utility values^32^;
5. excluding QALYs in the year of censoring/withdrawal;
6. discounting QALYs (3.5% per annum);
7. 1-y time horizon;
8. 3-ytime horizon;
9. model life-years as a biased estimate of trial QALYs (base-case assumptions); and
10. model QALYs as a biased estimate of trial life-years (base-case assumptions).

We also graphically compared predicted and observed cumulative incidence for individual events in participants with no baseline history of that event to compare our results against those of previous validation studies, explore whether prediction accuracy varied between events, and assess which endpoints require recalibration. Observed cumulative incidence was plotted for each individual event adjusting for death as competing risk using the stcompet command in Stata version 17 (StataCorp, College Station, TX, USA). This was compared graphically against the mean cumulative incidence predicted by UKPDS-OM1/UKPDS-OM2, estimated as the number of events predicted over time, divided by the number of individuals at start of simulation (Appendix 1).

## Results

### QALYs

**Figure 2:**
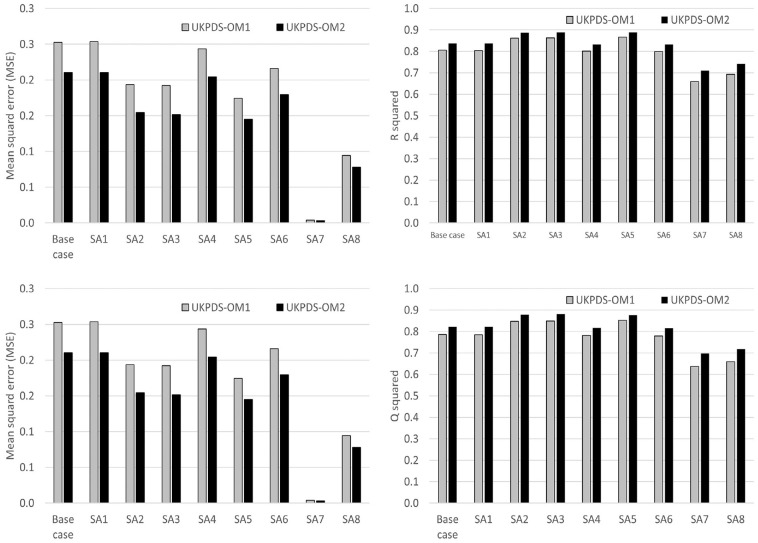
MSE, MAE, Q^2^, and *R*^2^ for QALYs. IHD, ischemic heart disease; MAE, mean absolute error; MI, myocardial infarction; MSE, mean squared error; Q^2^ = 1 − MSE/standard deviation^2^; QALY, quality-adjusted life-year; SA, sensitivity analysis; UKPDS-OM1, United Kingdom Prospective Diabetes Study Outcomes Model version 1; UKPDS-OM2, United Kingdom Prospective Diabetes Study Outcomes Model version 2. SA1: Trial QALYs include second MI, stroke, amputation, blindness, and ulcer since randomization regardless of patient history. SA2: Excluding disutility from second MI, stroke, or amputation in UKPDS-OM2: 8,269 patients with no prior MI, stroke, or amputation. SA3: Excluding ulcer and second events from UKPDS-OM2 (which are not captured in UKPDS-OM1): 8,269 patients with no prior MI, stroke, or amputation. SA4: Alternative utility values for both UKPDS-OM1 and UKPDS-OM2: initial utility, 0.785; IHD, −0.09; MI, −0.055; stroke, −0.164; heart failure, −0.108; blindness, −0.074; ulcer, −0.170; amputation, −0.280; renal failure, −0.204; disutility for subsequent years same as year of event.^32^ SA5: Excluding QALYs in the year when patients were censored for both trial and model QALYs. SA6: Discounting QALYs at 3.5% per annum. SA7: 1-y time horizon. SA8: 3-y time horizon.

**Figure 3:**
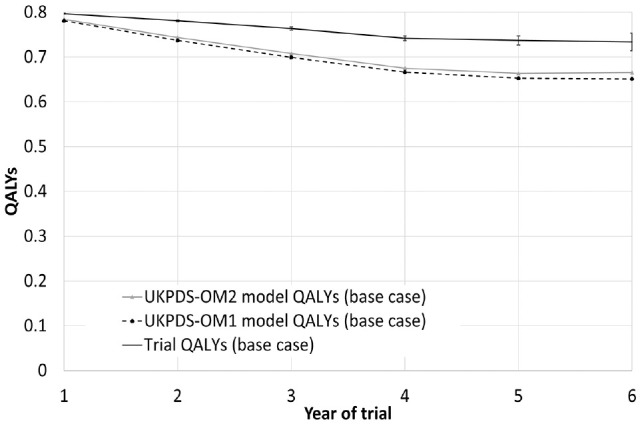
Trial and model QALYs in each year of the study for the base-case analysis. Person-years in which participants were censored or had been censored previously are excluded, although person-years after death are included. Error bars show 95% confidence intervals around trial QALYs. QALYs, quality-adjusted life-years; UKPDS-OM1, United Kingdom Prospective Diabetes Study Outcomes Model version 1; UKPDS-OM2, United Kingdom Prospective Diabetes Study Outcomes Model version 2.

The base-case analysis demonstrated that UKPDS-OM2 had better prediction accuracy for QALYs than UKPDS-OM1 (Figure 2, Table 1). The MSE for QALYs for UKPDS-OM2 was 17% lower than that for UKPDS-OM1, and MAE was 8% lower. Although both models had a downward bias, UKPDS-OM2 underestimated trial QALYs by an average of 0.127 (5.0%), while UKPDS-OM1 underestimated QALYs by 0.150 (5.8%; Table 1, Figure 3). Model QALYs for both models lay outside the 95% confidence interval [CI] of trial QALYs for each year (Figure 3). QALYs for both models mirrored the bimodal distribution of QALYs (Figure S5.1, Supplementary Material), but model QALYs had a lower standard deviation than trial QALYs did (Table 1).

**Table 1:** Results for the Base Case Analysis, Sensitivity Analyses Testing Performance Metrics, and Subgroup Analyses^a^

Analysis	Model	Trial QALYs, $\bar{x}$ (*s*)	Model QALYs, $\bar{x}$ (*s*)	Q^2^	*R* ^2^	MAE	MSE	Bias	*n*
Model QALYs v. model QALYs (base case)	OM1	2.573 (1.087)	2.423 (0.953)	0.786	0.805	0.289	0.253	−0.150	14,729
	OM2	2.573 (1.087)	2.445 (0.951)	0.822	0.837	0.265	0.210	−0.127	14,729
Model life-years v. trial life-years	OM1	3.226 (1.352)	3.028 (1.190)	0.797	0.819	0.342	0.372	−0.199	14,729
	OM2	3.226 (1.352)	3.058 (1.188)	0.829	0.846	0.314	0.313	−0.168	14,729
Extreme sensitivity analyses testing performance metrics									
Model life-years v. trial QALYs	OM1	2.573 (1.087)	3.028 (1.190)	0.596	0.809	0.522	0.477	0.455	14,729
	OM2	2.573 (1.087)	3.058 (1.188)	0.609	0.840	0.517	0.461	0.485	14,729
Model QALYs v. trial life-years	OM1	3.226 (1.352)	2.423 (0.953)	0.423	0.814	0.887	1.056	−0.803	14,729
	OM2	3.226 (1.352)	2.445 (0.951)	0.462	0.841	0.865	0.984	−0.781	14,729
Subgroup analyses by participant characteristics at randomization									
Age <65 y	OM1	2.630 (1.098)	2.568 (1.010)	0.879	0.883	0.184	0.145	−0.061	8,500
	OM2	2.630 (1.098)	2.555 (1.005)	0.886	0.892	0.186	0.137	−0.075	8,500
Age ≥65 y	OM1	2.495 (1.066)	2.225 (0.830)	0.649	0.718	0.433	0.399	−0.270	6,229
	OM2	2.495 (1.066)	2.296 (0.849)	0.727	0.768	0.373	0.311	−0.200	6,229
No prior MI, IHD, or stroke	OM1	2.694 (1.129)	2.570 (1.015)	0.840	0.853	0.241	0.204	−0.125	8,188
	OM2	2.694 (1.129)	2.595 (1.023)	0.865	0.874	0.216	0.172	−0.100	8,188
Prior MI, IHD, or stroke	OM1	2.421 (1.011)	2.240 (0.834)	0.693	0.726	0.349	0.313	−0.181	6,541
	OM2	2.421 (1.011)	2.258 (0.815)	0.747	0.778	0.327	0.259	−0.162	6,541
<5 y diabetes duration	OM1	2.658 (1.155)	2.568 (1.060)	0.847	0.853	0.218	0.204	−0.090	2,712
	OM2	2.658 (1.155)	2.568 (1.046)	0.863	0.870	0.216	0.182	−0.090	2,712
≥5 y diabetes duration	OM1	2.554 (1.070)	2.390 (0.925)	0.770	0.794	0.305	0.263	−0.163	12,017
	OM2	2.554 (1.070)	2.418 (0.926)	0.810	0.829	0.276	0.217	−0.136	12,017

IHD, ischemic heart disease; MAE, mean absolute error; MI, myocardial infarction; MSE, mean squared error; *n*, number of trial participants included in this analysis; OM1, United Kingdom Prospective Diabetes Study Outcomes Model version 1; OM2, United Kingdom Prospective Diabetes Study Outcomes Model version 2; QALY, quality-adjusted life-year; Q^2^ = 1 − MSE/S^2^; QALYs, quality-adjusted life-years; SD, standard deviation; $\bar{x}$ , mean; (s), standard deviation.

a For bias, zero indicates no bias, positive (negative) values indicate that the model overestimates (underestimates) trial QALYs, and larger absolute values indicate higher levels of bias.

Q^2^, which allows for bias in absolute values as well as the proportion of variability explained by the model, suggested that the proportional error reduction for UKPDS-OM2 was 82%, compared with 79% for UKPDS-OM1. UKPDS-OM2 model QALYs explained 84% of the variability in trial QALYs (coefficient of determination, *R*^2^), compared with 81% for UKPDS-OM1 (Figure 2).

**Figure 4:**
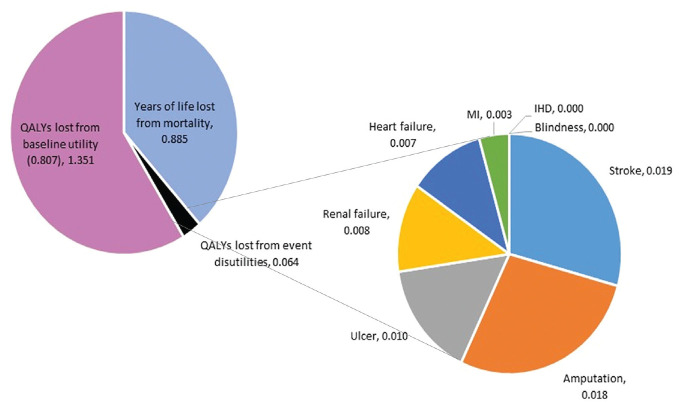
Breakdown of model QALYs for UKPDS-OM2 by event: quality-adjusted life-years (QALYs) lost through the baseline disutility associated with diabetes, QALYs lost through the disutilities attached to each event, and years of life lost through mortality before year 7. The impact of each individual event includes only the disutilities directly applied to that event: it excludes any mortality from fatal events of that type (which are counted in the years of life lost) and the impact of one event on the risk of another event. For example, the impact of stroke captures only the quality-of-life reduction from nonfatal stroke (0.165): the QALYs lost from fatal stroke and the QALYs lost from stroke survivors having higher mortality are counted in years of life lost, while the QALYs lost from MI and amputation (which occur at a higher rate following a stroke^4^) are counted under MI and amputation. IHD, ischemic heart disease; MI, myocardial infarction; QALYs, quality-adjusted life-years; SA, sensitivity analysis; UKPDS-OM2, United Kingdom Prospective Diabetes Study Outcomes Model version 2.

Mortality was the main driver of model QALYs: only 0.064 QALYs were lost through quality-of-life reductions associated with diabetic events versus 0.885 QALYs lost through mortality (Fig. 4). Stroke and amputation reduced QALYs more than all other events combined.

### Subgroup and Sensitivity Analyses

UKPDS-OM2 had lower MSE and higher Q^2^ than UKPDS-OM1 in all subgroup analyses (Table 1). However, UKPDS-OM1 had lower MAE than UKPDS-OM2 and a bias closer to 0 for participants aged <65 y. Both models had lower Q^2^ for older people, those with prior cardiovascular disease, and those with longer diabetes duration compared with the overall population. MSE and MAE cannot be directly compared between analyses or subgroups as they depend on the variability in trial QALYs.

Varying the methods in sensitivity analysis had very little impact on Q^2^ (Figure 2). UKPDS-OM2 outperformed UKPDS-OM1 for all performance metrics in every sensitivity analysis. Q^2^ for life expectancy was similar to Q^2^ for QALYs (Table 1). Comparing model versus trial life expectancies rather than QALYs had similar Q^2^ to the base-case analysis (Table 1).

Two sensitivity analyses evaluating extremely biased models were conducted to assess the extent to which different performance metrics pick up biased estimators: model life-years systematically overestimate trial QALYs, and trial QALYs systematically underestimate model life-years. The *R*^2^ for these scenarios was similar to that for the base-case analysis, despite the substantial biases (Table 1). By contrast, Q^2^ was substantially lower and MSE was substantially higher, reflecting the substantial bias.

### Cumulative Incidence of Events

**Figure 5:**
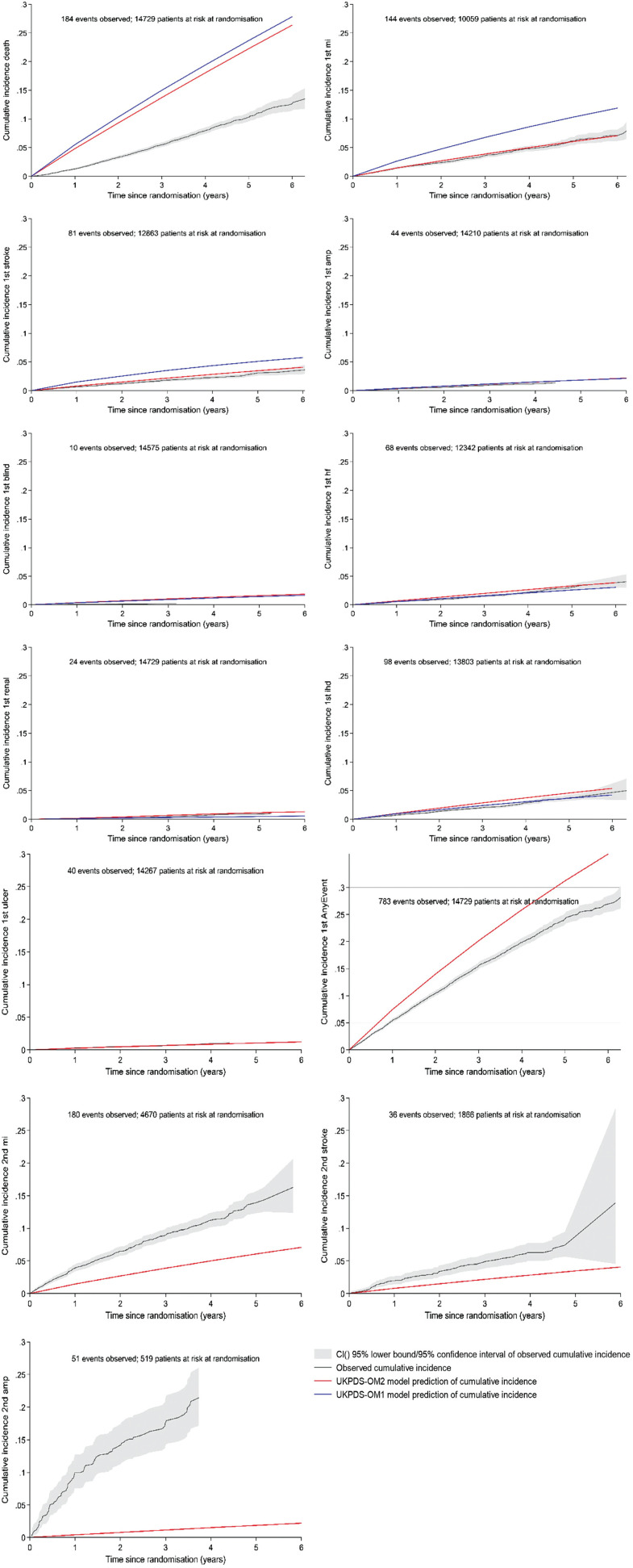
Observed (95% CI) cumulative incidence and predicted cumulative incidence from UKPDS-OM1 (blue line) and UKPDS-OM2 (red line) for individual events in the base-case analysis. amp, amputation; AnyEvent, first event of any type; CI, confidence interval; IHD, ischemic heart disease; MI, myocardial infarction; HF, heart failure; MSE, mean squared error; QALY, quality-adjusted life-year. Ulcer, second events, and the composite endpoint any event cannot be estimated by UKPDS-OM1 and so are shown only for UKPDS-OM2. The observed cumulative incidence of amputation, blindness, renal failure, and ulcer is plotted up to the last occurrence of that event in the trial. Deaths and any event are based on all patients. All other graphs plotting the incidence of the first event of each type are plotted only for the subset of patients who had no history of that event at randomization; graphs for second events are plotted only for patients who had a history of that event at randomization.

Plotting the cumulative incidence for individual events demonstrated that both models overestimate the incidence of death, first MI, first stroke, and blindness (Figure 5). UKPDS-OM2 performed better for mortality, MI, and stroke, while UKPDS-OM1 performed better for blindness and IHD. The predictions of both models partially overlapped the 95% CIs of the observed data for amputation, heart failure, and renal failure; for heart failure and renal failure, UKPDS-OM2 overestimated the incidence, while UKPDS-OM1 underestimated incidence. UKPDS-OM2 gave good predictions of ulcer and overestimated the incidence of second MI, second stroke, and second amputation, which are not included in UKPDS-OM1. UKPDS-OM2 also overestimated the first occurrence of any event (including death).

## Discussion

QALYs can be used as an informative global outcome to assess prediction accuracy for individual patient simulation models used for HTA and economic evaluation, since they capture the occurrence of multiple clinical events in a single outcome that is relevant to that application. QALYs give the greatest weight to deaths and clinical outcomes associated with the largest quality-of-life decrements. They allow for the timing of events, giving greater weight to deaths or irreversible conditions that occur early in the study period and giving greater weight to common events than rare events.

The prediction accuracy for QALYs gives limited information about which event(s) are predicted well or poorly. Theoretically, a model could have good prediction accuracy for QALYs (or clinical composite endpoints) despite one event being overestimated and another being underestimated if both events arose in patients with the same characteristics. Using QALYs alongside additional analyses, such as cumulative incidence curves, can help overcome these shortcomings and identify which parameters need recalibration.

Prespecifying a single primary outcome in an analysis plan can help reduce reporting bias.^11^ The primary outcome should reflect the application for which the model will be used. If a single global outcome is needed and the model is used for economic evaluation, QALYs could be a candidate that is likely to be more informative than composite outcomes such as major cardiovascular events.

In the EXSCEL sample, UKPDS-OM2 produced more accurate and less biased predictions than UKPDS-OM1 for QALYs and mortality. This extends a previous study by Hayes et al.,^4^ who observed that in the original UKPDS sample, UKPDS-OM2 predicted fewer cardiovascular events and deaths than UKPDS-OM1 did. It is likely that part of the difference between the models is due to secular trends in mortality and management of diabetes and diabetic events, since UKPDS-OM2 includes more recent data. Internationally, mortality rates and the incidence of diabetic events have dropped sharply since the UKPDS study began in 1977.^33^ UKPDS-OM2 was also estimated using more data and incorporated additional risk factors and second events. Our conclusions about which model was best were sensitive to the outcome used to estimate prediction accuracy, with UKPDS-OM1 giving better predictions for amputation, blindness, and IHD and UKPDS-OM2 performing better for QALYs and mortality. This highlights the importance of choosing the primary measure carefully.

Model and trial QALYs depend on the assumptions that are used to estimate the impact of events on quality of life. In this analysis, we followed the assumptions used to calculate QALYs in UKPDS-OM2 (Appendix 3). This may have contributed to the better prediction accuracy for UKPDS-OM2. One such assumption was that second MI, stroke, and amputation have the same impact as the first event of that type, but that third MI, stroke, or amputation or second ulcer have no further impact. In principle, trial QALYs could be estimated with fewer assumptions to test other model assumptions, such as the half cycle correction. Our approach could also be extended to evaluate uncertainty measures around model estimates,^28^ allowing for both parameter and sampling uncertainty.^34^

UKPDS-OM2 performed reasonably well in the EXSCEL sample but overestimated the incidence of death and most first events, while underestimating QALYs and second events. It is likely that further recalibration will be needed to accurately predict events and QALYs in contemporary global populations, particularly for second events.

EXSCEL provided a large contemporary international population, recruited patients with a wide range of risk factor values, and adjudicated clinical outcomes. The EXSCEL and UKPDS populations differ in that UKPDS recruited patients with newly diagnosed diabetes,^22^ whereas EXSCEL participants were diagnosed a median of 12 y before randomization and 73% had prior cardiovascular events.^24^ However, EXSCEL has limitations for external validation. First, the median follow-up was only 3.2 y, whereas economic evaluation generally requires a lifetime horizon.^13^ There are very few real-world datasets with data on all UKPDS-OM risk factors that have longer follow-up; registry studies often include only risk factor measurements that are indicated by patients’ symptoms (introducing bias) and will not have adjudicated clinical outcomes. Second, EXSCEL (like many diabetes trials) excluded participants with frailty, dementia, or life expectancy <2 y, which may mean that event rates and mortality in EXSCEL are lower than for routine clinical practice. Consequently, the standardized mortality ratio (SMR) for US patients in EXSCEL compared with the 2015 US general population^35^ was 0.75 in the patients’ first year after randomization and 1.35 in year 3, whereas registry studies observe higher SMRs (e.g., 1.38 for men and 1.49 for women^36^). While the models overestimated mortality in EXSCEL, it is less clear whether they would overestimate mortality in “typical” diabetes populations. Third, some events were defined differently from UKPDS (Appendix 1, Table S1): EXSCEL recorded hospitalization for heart failure/unstable angina and gangrene rather than diagnosis of heart failure/IHD and ulcer, so the actual incidence of these may be higher. Finally, EXSCEL did not collect data on white blood cell count or postrandomization smoking.

One disadvantage of QALYs is the need to choose a set of utility values for events. However, sensitivity analyses showed that prediction accuracy was not very sensitive to utilities. The base-case analysis assigned no disutility to IHD or blindness, since these had no significant effect on utility in UKPDS.^27^ The accuracy with which these events were predicted therefore had no effect on MSE for QALYs (except insofar as participants with IHD are at higher risk of mortality or other cardiovascular events). However, any composite endpoint and any approach to assessing global prediction accuracy will inevitably rely on weights of some kind, and we are not aware of any other set of weights with a better evidence base.

In principle, costs of different events could be used as an alternative set of weights, whereby different events accrue different costs and models are compared based on the accuracy with which total costs are predicted. Prediction accuracy for costs could be important if high-cost nonfatal events are poorly predicted. However, fatal events generally have low cost, and patients who die accrue no further costs: using costs as a global measure of prediction accuracy could favor models that poorly predict mortality, which is one of the most important model outcomes. Poor prediction of fatal and nonfatal events could also cancel out and erroneously suggest a model produced good predictions. Costs are also likely to vary between countries and over time more than utility weights, which may introduce challenges for generalizability, especially in multinational studies. Furthermore, as most diabetes treatments are taken for a lifetime, the impact of prediction accuracy on total cost depends heavily on treatment cost, which will vary between applications or model arms. By contrast, it is necessary to choose a single model that can be applied to both intervention and control and often (e.g., for multinational studies) for multiple countries. Net benefit could also be used: this would combine prediction accuracy for both cost and QALYs but would be sensitive to assumptions about setting, treatment cost, and ceiling ratio. We therefore consider QALYs a more generalizable outcome for assessing global model performance.

One challenge for comparing prediction accuracy for QALYs is that different simulation models include different events and apply the quality-of-life impact of events differently (e.g., additive or multiplicative). However, this is not a challenge if we are assessing the impact of recalibrating a single model. Furthermore, some datasets do not provide data on all events, which means that we would be able to assess the impact of the reported events only on QALYs, which may lead to overestimation of both model and trial QALYs. However, our approach does not require any more data than validating each event individually. The code for estimating QALYs is provided in Appendix 2.

Our analysis methods are illustrated by validating 2 closely related diabetes microsimulation models using a single trial. However, the same approach could be applied to any model able to simulate outcomes for a population of patients based on their baseline characteristics, including many cohort models (e.g., Schlackow et al.,^37^ Dakin et al.,^38^ Heart Protection Study Collaborative et al.,^39^ and Stevenson et al.^40^). Future research to evaluate our methods in other disease areas would be valuable. We used the EQ-5D values built into UKPDS-OM2, but our methods could be used with any set of utility (or disability) weights, reflecting the preferences of either the general public or patients.

To our knowledge, this article is the first to use Q^2^ (1 minus MSE divided by standard deviation squared)^18,19^ in health economics. This provides an absolute measure of prediction accuracy that can be compared between outcome measures and between samples. Q^2^ has all of the advantages of MSE: it captures discrimination, imprecision, and bias and penalizes models more for larger prediction errors than smaller prediction errors. *R*^2^ captures only discrimination, and a biased model may have a high *R*^2^, whereas Q^2^, MSE, and MAE capture both discrimination and bias. Bias is likely to be particularly relevant to population-level cost-effectiveness. However, while MSE values indicate only relative performance and cannot be compared between outcomes or between samples, Q^2^ can be interpreted in a similar way to *R*^2^. Q^2^ is also more sensitive to outliers than *R*^2^ is (which in this case could be low-risk participants who died early in the trial and patients with long follow-up) and penalizes econometric models for collinearity more than *R*^2^.^18^

In conclusion, QALYs can be used as an outcome measure when assessing prediction accuracy of decision-analytical models that predict outcomes for individual patients. Similar methods could be applied to models of other diseases that can predict outcomes for individual patients. Q^2^ could be used for any application in which MSE is used, including mapping models, prognostic models predicting continuous endpoints, and any econometric application.

## Supplemental Material

sj-do-2-mdm-10.1177_0272989X241285866 – Supplemental material for Using QALYs as an Outcome for Assessing Global Prediction Accuracy in Diabetes Simulation Models Supplemental material, sj-do-2-mdm-10.1177_0272989X241285866 for Using QALYs as an Outcome for Assessing Global Prediction Accuracy in Diabetes Simulation Models by Helen A. Dakin, Ni Gao, José Leal, Rury R. Holman, An Tran-Duy and Philip Clarke in Medical Decision Making

sj-docx-1-mdm-10.1177_0272989X241285866 – Supplemental material for Using QALYs as an Outcome for Assessing Global Prediction Accuracy in Diabetes Simulation Models Supplemental material, sj-docx-1-mdm-10.1177_0272989X241285866 for Using QALYs as an Outcome for Assessing Global Prediction Accuracy in Diabetes Simulation Models by Helen A. Dakin, Ni Gao, José Leal, Rury R. Holman, An Tran-Duy and Philip Clarke in Medical Decision Making
