# Supplementary material for: Using QALYs as an Outcome for Assessing Global Prediction Accuracy in Diabetes Simulation Models
Source: Med Decis Making. 2024 Oct 30;45(1):45–59. doi: 10.1177/0272989X241285866 (PMC11645849; doi:10.1177/0272989X241285866)
Supplement: sj-docx-1-mdm-10.1177_0272989X241285866 – Supplemental material for Using QALYs as an Outcome for Assessing Global Prediction Accuracy in Diabetes Simulation Models [file sj-docx-1-mdm-10.1177_0272989X241285866.docx]

**Supplementary material for “Using QALYs as an outcome for assessing global prediction accuracy in diabetes simulation models”**

**Submitted to *Medical Decision Making***

**Appendix 1: Methods for imputation, data preparation and UKPDS-OM model set up**

**Figure S1.1:** Flowchart of study


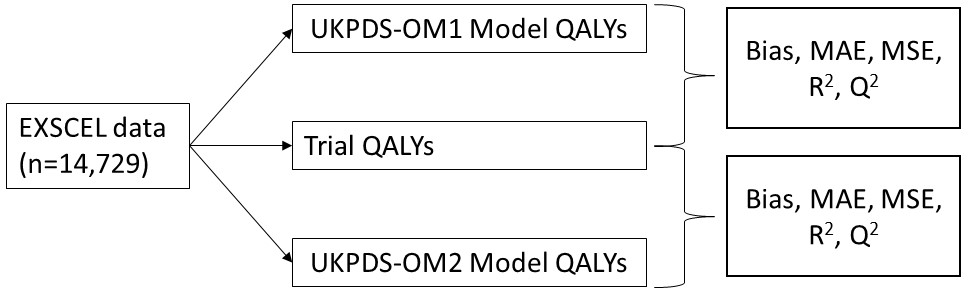


Abbreviations: MAE, mean absolute error; MI, myocardial infarction; MSE, mean squared error; QALY, quality-adjusted life-year; Q^2^ =1-MSE/SD^2^; QALYs, quality-adjusted life-years.

***Inclusion criteria***

We included all patients who were randomised and for whom there were any data on risk factors and ≥1 day of follow up for cardiovascular events. We did not apply any additional exclusion criteria in order to ensure that we maximise the diversity within our population.

The only exclusion criteria were:

- Patients who have missing data on at least 15 risk factors at baseline (including baseline history). The rationale for this is that we cannot generate any meaningful predictions of life expectancy or cardiovascular risk in this population.
- Patients for whom the follow-up is less than one day. Patients who withdrew on the same day as randomisation were therefore excluded from the analysis because they have no data to inform event rates.
- Patients with missing data on ethnicity were excluded. This is because it was not feasible to impute such data with multiple imputation in a multinational trial because ethnicities vary substantially between countries and a highly complicated imputation function would be needed to impute ethnicity data in a valid way.

***Event definitions***

**Table S1.1:** Definitions of risk factors & events that differ between datasets. Information on UKPDS definitions have been adapted from the supplementary appendix 3 of Hayes et al 20213.**^1^**

|  | **UKPDS^1^ (supplementary appendix 3)** | **EXSCEL^2^ (supplementary appendix page 14-26)** |
| --- | --- | --- |
| MI | MI (first or subsequent), defined as acute myocardial infarction (ICD-9 code 410) or sudden death (ICD-9 code 798 and 798.9)* | Baseline history: myocardial infarction  Clinical events: Since previous visit, has the patient experienced any of the following: myocardial infarction* |
| Stroke | Stroke (first or subsequent), defined as non-fatal stroke (ICD-9 code 430 and 434.9, or 436) or fatal stroke (ICD-9 code 430 and 438.9).* | Clinical events: Since previous visit, has the patient experienced any of the following: Cerebrovascular disease: stroke* |
| IHD | Other chronic ischemic heart disease (IHD), defined from ICD-9 code 411 and 414.9* | Unstable Angina Requiring Hospitalization Baseline_Med_Hx_Unstable_Angina, IHD_Date  Clinical events: Since previous visit, has the patient experienced any of the following: Cerebrovascular Disease: >/= 50% stenosis of carotid artery  Clinical events: Unstable Angina/Recurrent Ischemia requiring hospitalization* |
| Heart failure | Congestive heart failure (CHF) defined as fatal or non-fatal ICD-9 code 428 and 428.9. * | Congestive heart failure requiring hospitalisation. Clinical events: Was the patient admitted to an inpatient unit for CHF?* |
| Ulcer | Ulcer of the lower limb (ICD-9 codes 707.1, 707.14, 707.15 or 707.19)* | Baseline history: foot ulcer  Post-baseline: gangrene |
| Renal failure | Renal failure defined as creatinine >250 mol/l not ascribable to any acute intercurrent illness (ICD-9 codes 250.3 and 585 and 586) and death due to renal failure (ICD-9 codes 580 and 593.9)* | Since the previous visit, did the patient experience any new or worsening occurrences of any of the following: End-stage Renal Failure needing chronic Peritoneal/Hemodialysis (including creation of fistula or other vascular access for hemodialysis) or renal transplantation. If criteria not met, answer question NO. |
| Blindness | Blindness in one eye defined as a visual acuity Snellen 6/60 or ETDRS logMAR 1.0 or worse for any reason, persisting for 3 months (ICD-9 codes 369 and 369.9)* | Baseline history: blindness  Since the previous visit, did the patient experience any new or worsening occurrences of any of the following: blindness |
| Amputation | Limb amputation as amputation of at least one digit.  Amputation (first or subsequent) of a digit or limb (ICD-9 code 5.845 and 5.848, or 250.6) or a fatal peripheral vascular event (ICD-9 code 997.2, 997.6, 250.6 or 440.2)* | Baseline history: amputation  Since the previous visit, did the patient experience any new or worsening occurrences of any of the following: amputation.  Before analysis, we counted amputations within 28 days and those reported on the same date with missing event dates as a single amputation (not as 2 amputations). |
| PVD | Presence of intermittent claudication or ankle brachial pressure index <0.9* | Since the previous visit, did the patient experience any new or worsening occurrences of any of the following: >= 50% stenosis of lower extremity artery or ABI <0.9 (PAD) |
| Albuminuria | Presence of macro or micro albuminuria | Baseline history: albuminuria (micro, macro, unknown)  Since the previous visit, did the patient experience any new or worsening occurrences of any of the following: Albuminuria |
| Timing of event history | Events before diabetes diagnosis were not taken into account | Baseline history of events could include events before diagnosis of diabetes. When setting up the model, we assumed that all events occurred >12 months before the start of the trial. |

***Data cleaning***

- If only year of diagnosis, year of event or year of birth were available, we assumed that diagnosis/birth/event/randomisation/censoring occurred on 30^th^ June of that year (halfway through the year). If we only had year and month, we assumed that the diagnosis/birth/event/randomisation/censoring was on the 15^th^ of that month (halfway through the month). This follows the methods used to impute missing day or month of birth when estimating age in the main analysis of EXSCEL clinical outcomes.
  - If a patient was known to have an event, but the date of this event is completely missing, the event was assumed to have occurred on the date when the event was reported is given providing this is available.
- If only the year of diagnosis of diabetes was recorded, the duration was estimated by subtracting the year of diagnosis of diabetes from the calendar year in which randomisation took place; for patients who were randomised in the year that they were diagnosed, the duration of diabetes was set to 0.25 years, since one of the inclusion criteria of EXSCEL is that patients had to be unstable antidiabetic medication for at least three months.
- Implausible values: any values that were out of the range accepted by UKPDS-OM were treated as missing and those values were imputed using multiple imputation.
- When there was more than one continuous risk factor measurement per year, we took the average across the measurements taken in that year. For example, EXSCEL measured risk factors every 6 months: Year 1 HbA1c equalled the average of measurements at 6 and 12 months and Year 2 equalled the average of 18 and 24 months post-randomisation. If 1 of these values was missing, we took the average over the remaining values for that year. This minimised measurement error and regression to the mean, increased the stability of risk factor values, mirrored the methods used to estimate risk equations in UKPDS and reduced the amount of missing data. We assumed that all visits occurred at the scheduled time: for example we assumed that year 1 equalled the average of visit 2 (6 months) and visit 3 (12 months) and year 2 equalled the average of visit 4 (18 months) and visit 5 (24 months), regardless of how many visits patients had; we only included data on each patient's first 17 visits and any subsequent visits were not used in any analysis. This process was done before multiple imputation in order to (a) reduce the amount of missing data; and (b) ensure that the imputation model matches the analysis as closely as possible.
- If we had the exact date of onset for albuminuria, PVD and atrial fibrillation, we assumed that patients had this condition at year 1 if the date of onset is <365.25 days after randomisation. Patients were considered to have the risk factor in year y if they have a recorded instance of that event at baseline or within y*12 months of randomisation and were considered to not have the event if they did not have any cases of that event within y*12 months of randomisation, regardless of whether they were observed for the full year. For example, a patient who withdrew at three months without having been diagnosed with PVD, AF or albuminuria was considered to have none of these events at year 1. However, trajectories were used to impute values for any year in which the patient was not observed at all. This assumption was made to use the data that was available from partially-observed years and simplify the analysis.
- Since EXSCEL did not collect data on the date of events that occurred before randomisation, all pre-randomisation cardiovascular events were included in patients’ baseline history regardless of whether they occurred before or after the diagnosis of diabetes. When the original UKPDS equations were estimated and in at least one validation study^3^, cardiovascular events before the diagnosis of diabetes were excluded and all patients were assumed to have had no history of events at the beginning of the study. Since the mean duration of diabetes in EXSCEL was 12 years,^4^ it is highly likely that the vast majority of pre-randomisation events occurred after the diagnosis of diabetes.
- Events occurring before randomisation were not considered events within any analysis recalibrating the model or estimating model validity or prediction accuracy but were instead counted in patients’ baseline history.
- The observed data on clinical events for each patient was considered to be censored at the last time point when reliable data on clinical events exist for that patient. Detailed methods for how this was identified are given in the next section.
- In any cases where a patient had an event on the day of randomisation, but after they were randomised, this event was assumed to occur 0.5 days after randomisation to ensure that this participant was not dropped from survival analyses.
- The date of the censoring for individual events in EXSCEL was generally equal to the end of study date for patients who didn’t have the event, but in a number of cases, the censoring date for non-fatal events was earlier than the end of study date because the status check for that event happened to be earlier than the status check for death. In these cases, generally the censoring date was the same for most of the adjudicated events. The events that are adjudicated in EXSCEL are the primary composite endpoint (PCO), MI, stroke, CHF and angina (HUA). For example, hypothetical patient 1 may have an end of study date of 31/12/2017, had a non-fatal stroke on 1/11/2016 and dAMI= 30/05/2017, dHHF= 30/05/2017, dHUA= 30/05/2017.
  - In the analyses of individual adjudicated first events (e.g. estimating prediction accuracy for first MI), we used the date of event/censoring for that individual event (e.g. dAMI) as the date of censoring. For example, for patient 1, the date of censoring for MI and heart failure would be 30/05/2017.
  - For analyses specifically estimating mortality, the date of censoring was the end of study date, because the end of study date represents the status check for mortality. For example, for patient 1, the date of censoring for mortality would be 31/12/2017.
  - If censoring date for specific event date > study end date, we used study end date and excluded any events after end of study date because we are unlikely to have unbiased and complete follow up of all events after patient leaves the trial.
  - In analyses of unadjudicated events, second events and QALYs, we combined data on the censoring date for adjudicated events to get a single corrected end of study date. For EXSCEL, the common censoring date was calculated as the event date (where it occurs) or the earliest of the right censor date (RTCNSDT) and the last event assessed date (LEVASDT).
  - For IHD, there were 204 patients in EXSCEL where the patient has 0 in the IHD column but the patient later has an event on the subsequent events dataset before the common censoring date; in these cases, the censoring date for IHD often equals the date of the primary endpoint. In these cases only, we treated the date of the first IHD event on the “subsequent events” dataset as the date of IHD diagnosis and set the IHD variable to equal 1.
  - In EXSCEL, there were a significant number of patients who had a large number amputations: e.g. reporting two amputations at one visit, two more at their study visit six months later and, three years later, reported four more amputations (eight amputations in total; dates have been changed). By contrast, in the UKPDS trial dataset, the mean time between first and second amputation was two years and the minimum time was five days, but most amputations were a lot further apart. Multiple amputations during one hospital stay most likely reflect initial conservative management efforts that were unsuccessful. We therefore excluded (a) any amputations that occurred <28 days after another as a single amputation event and (b) any amputations for which we have missing event date and a report date that is shared with other amputations (based on the assumption that this amputation probably occurred in the same hospital stay as other amputations). For the example above, the patient would be considered to have four amputations (or sets of amputation).

***Missing data and imputation***

Since cardiovascular events comprised the primary EXSCEL outcome measure, event history was complete for the full duration of each participant’s follow-up. We therefore assumed that we have complete data on cardiovascular and diabetic events (including atrial fibrillation and PVD) up until the point until patients are censored.

We used a combination of multiple imputation of risk factors at baseline in year 1 and risk factor trajectories^5, 6^ to predict risk factors from year 2 onwards. This approach avoids excluding large numbers of patients who are missing data on only 1 or 2 risk factors and to minimise any bias that would have been introduced by excluding patients with data not missing completely at random. This also minimises the extent to which we need to rely on the risk factor trajectories built into the model.

We used risk factor trajectories for continuous risk factors (HDL-cholesterol, LDL-cholesterol, HbA1c, haemoglobin, heart rate, blood pressure and body mass index) estimated based on EXSCEL and TECOS by Gao et al 2024.^5^ We used the trajectories for estimated glomerular filtration rate, peripheral arterial disease, atrial fibrillation, albuminuria, smoking and white blood cell count that were developed by Leal et al^6^ since these are built into UKPDS-OM version 2.2.^7^

This approach was used as it will ensure that missing values for risk factors are broadly in line with that patient's other risk factor values. Generally, risk factor values for individual patients are fairly stable over time or creep gradually in a particular direction and the risk factor trajectories will mirror this. By contrast, multiple imputation could theoretically impute quite different values at certain time points: particularly in later years when there is less data due to administrative censoring.

Our analysis used data from 14,729 participants after excluding 23 participants for whom there was insufficient data for the analysis. We imputed missing data on cardiovascular risk factors at baseline and Year 1 using multiple imputation. Baseline and year 1 triglycerides were imputed using mean imputation as these were only needed for UKPDS-OM1. We used risk factor trajectory equations estimated on EXSCEL and the Trial Evaluating Cardiovascular Outcomes with Sitagliptin (TECOS) trial to impute missing data on continuous risk factors subsequent years,^5^ while trajectories for binary risk factor and glomerular filtration rate were based on published equations.^6^ Missing data on total cholesterol was estimated using the trajectories built into UKPDS-OM1.^8^ Since white blood cell count was not measured in EXSCEL, we imputed baseline values from other risk factors using the equations by Pagano et al^3^ and extrapolated these using published risk factor trajectories.^6^

Since participants had to be on stable treatment at randomisation and there were no data on the timing of historical events, we assumed that all pre-randomisation events occurred ≥12 months earlier. The same assumption has been used previously.^9^

We did multiple imputation of each patient’s baseline and year 1 risk factor data to ensure that each patient has at least one value for each risk factor during the post-treatment period. For randomised trials, it is recommended that mean imputation is done for baseline values to avoid having treatment effects introducing baseline imbalance between randomised groups.^10^ However, we did not do any such mean imputation here, because we are not interested in comparisons between randomised groups, are not using the baseline values for continuous risk factors in any analysis and do not want to systematically underestimate the variability between population groups through mean imputation.

Patients who withdrew on the day of randomisation and patients who were excluded on the basis of having missing ethnicity or missing data on >15 risk factors were dropped from the dataset before multiple imputation.

Multiple imputation included the following variables:

- Continuous risk factors at baseline and year 1
  - We imputed untransformed diabetes duration, height and weight and did not include transformations or BMI because mi impute does not passively impute these for each imputation.
  - eGFR was treated as a continuous variable without a spline or dummy variable to keep the imputation function simple and avoid perfect prediction. This deviates slightly from the UKPDS-OM, but the predictive mean matching will overcome most distributional issues.
- Treatment allocation: this was be included in the model as a predictor of year 1 risk factors, but not a predictor of any baseline continuous variable. Although our analyses did not consider the effect of study treatment, treatment allocation was included in the imputation model because it was expected to improve imputation estimates.
- Smoking and baseline binary risk factors with missing data (PVD in the case of EXSCEL) were included as imputed variables.
- Pre-randomisation history of cardiovascular/diabetic events (IHD, heart failure, stroke, MI, ulcer, blindness, amputation) and binary risk factors (PVD, AF & albuminuria) prior to baseline. Even if such events have complete data, these were included in the imputation model to help predict year 1 risk factors. Blindness was omitted as a predictor for EXSCEL due to perfect prediction.
- Dummies indicating whether patients have binary risk factors or cardiovascular events occurring during year 1 were also included in the imputation model because having cardiovascular events in the first year will increase the chance of a patient missing all post-baseline data AND increase the chances they have clinically poor risk factor data. Blindness was omitted from the imputation function to avoid perfect prediction. However, even if there were missing data on any of these events, the imputed values for these were not used in any subsequent analyses (PVD, AF & albuminuria at year 1 were based on events that occurred before the patient left the trial, while event data was assumed to be complete until censoring).
- Patients who died during year 1 were included in the imputation analysis and we imputed year 1 risk factor data even if patients died before the six-month follow-up. Mortality during year 1 was not included as a variable in the imputation model because it introduced convergence problems due to perfect prediction.
- Demographics with complete data:
  - Age, sex,
  - Life expectancy age 60 for the country the patient was recruited in. This life expectancy was based on that in 2015.
  - 5 ethnicity dummies: 1=Caucasian or white; 2=Black; 3=Asian (other); 4=Asian (Oriental); 5=Hispanic; baseline category=Other.
  - 5 geographical region dummies: countries were categorised into geographical regions using a classification modified from that used in the global burden of disease study (using the classifications described in Section 18)
    - Central and Eastern Europe and Central Asia and South Africa
      - South Africa was included in this category based on its life expectancy and GDP and because it is the only African country in EXSCEL
    - Asia other
      - We did not differentiate between South and East Asia because India was the only country for which we have data
    - Latin America and Caribbean region;
    - High-income regions excluding USA
    - USA: USA was dealt with separately because it accounts for such a large proportion of patients from high-income countries

Post-year 1 data was not included in order to keep the imputation model simple and avoid issues of convergence.

Predictive mean matching with 5 nearest neighbours was used to ensure that the imputed values are in range and follow a similar distribution.

***Post-imputation step:***

Albuminuria, PVD, atrial fibrillation were set to missing in the years after death if the patient died before these risk factors arise. Conversely, if 1 of these risk factors had already been diagnosed before death, that patient was assumed to have that risk factor indefinitely. This step ensured that values for these risk factors after the patient's actual date of death are imputed using trajectories (rather than assuming that the patient never develops those events in any loop).

We generated M datasets using multiple imputation (where M equals the percentage of patients who have any missing data) and then took the mean across the M datasets for each continuous risk factor risk factor for each patient at each time point (not propagating uncertainty into the analysis). For binary risk factors, we counted patients as having value 1 for that variable if the proportion of imputed datasets in which the patient is expected to have that event exceeds a cut off value. The best cut-off value was identified by varying the cut-off value across a wide range and selecting the cut-off value at which the proportion of patients with a 1 imputed for that binary risk factor equals the proportion of patients with value one among those who have non-missing values for that risk factor. Pagano et al. used the same approach^3^ and found that it gave very similar point estimates to propagating the uncertainty into the analysis. One implication of this approach is that averaging across the imputed datasets reduces the standard deviation of the imputed values because the averaging reduces stochastic uncertainty, but not heterogeneity. Based on this analysis using EXSCEL data, a cut-off of 0.2751 was estimated for baseline albuminuria, 0.5750 for baseline smoking, 0.1001 for baseline PVD and 0.1250 for baseline atrial fibrillation. For example, patients were considered to have albuminuria at baseline if ≥27.5% of imputations predicted them to have albuminuria. The same cut-offs were applied to data values beyond year 1 that were imputed using trajectories.

Patients’ pre-randomisation risk factor data were entered into the “inputs” worksheet of the UKPDS-OM, while the mean of 6 and 12 months was entered into the “year 1” column for each of the risk factors. Age, duration of diabetes and history of cardiovascular events was entered for the time of randomisation. For both baseline and year 1, missing data was equal to the mean across multiple imputations (see imputation section above).

Since participants had to be on stable treatment at randomisation and there were no data on the timing of historical events, we assumed that all pre-randomisation events occurred at least 12 months earlier. The same assumption has been used for previous analyses.^9^

Observed risk factor data from 18 months onwards was entered into the model on the worksheets showing the trajectories of each individual risk factor: the mean of 18 & 24 months was entered for year 1 and the mean of 30 and 36 months was entered for year 2.

Any gaps in risk factor data from year 1 to the follow-up time for the longest patient in that study were filled in by applying risk factor trajectories^5, 6^ to that patient’s most recent observed measurement (e.g. patients 2-3 in Table S1.2). If a patient has no observed year 1 data, we used the value imputed for year 1 (e.g. patients 4 & 5 in Table S1.2) and applied trajectories to estimate subsequent missing measurements; this was done even if the patient had data for subsequent years (e.g. patient 5), because the random effects model used by Gao et al^5^ and Leal et al^6^ can only project values forwards, not backwards.

This method was used to enter data after administrative censoring and after death because such data is required by the model. However, we only compare cardiovascular events up until the time when that patient reached the end of the trial.

**Table S1.2:** Example of how multiple imputation and trajectories were used to impute for missing data

| Pt | Y1 | Y2 | Y3 | Y4 | Y5 | Y6 | Y7 |
| --- | --- | --- | --- | --- | --- | --- | --- |
| 1 | Observed | Observed | Observed | Observed | Observed | Censored | Censored |
| 2 | Observed | Y1+d | Y2+d | Y3+d | Y4+d | Censored | Censored |
| 3 | Observed | Y1+d | Y2+d | Observed | Observed | Censored | Censored |
| 4 | Imputed | Y1+d | Y2+d | Y3+d | Y4+d | Censored | Censored |
| 5 | Imputed | Y1+d | Y2+d | Observed | Observed | Censored | Censored |
| 6: LA | Observed | Observed | Observed | Observed | Observed | MeanY5&Y7 | Observed |
| 7: LA | Observed | Observed | Observed | Observed | Y4+d | MeanY5&Y7 | Observed |

For WBC, which was not measured at all in EXSCEL, we used the equations that were estimated on the UKPDS trial and reported in Supplementary tables 5 and 6 of Pagano 2021^3^ to predict the unmeasured risk factors as a function of observed risk factor data. This was done for baseline data after multiple imputation. For future years, WBC values were imputed from baseline values using the risk factor trajectories estimated by Leal et al.^6^

The trajectory models estimated by Gao et al^5^ code ethnicity as: white; black; any Asian background (including oriental and Indian); and the baseline category of Hispanic/other (where other is a mix of Maori, aboriginal, Pacific Islander, Native American and only accounts for about 100 people). For continuous endpoints, we used all ethnicity categories and predicted missing values for the continuous endpoint based on each patient’s actual ethnicity.

***Set up of UKPDS-OM 2.1 for simulations***

The Model Parameters sheet of UKPDS-OM2 was set up using the following options for the base case analysis.

**Figure S1.2:** Set up of UKPDS-OM2 ‘Model Parameters’ sheet for the base case analysis. The inputs used in our simulation are presented using the text and format of the UKPDS-OM2.1 input spreadsheet developed by Oxford University.^7^


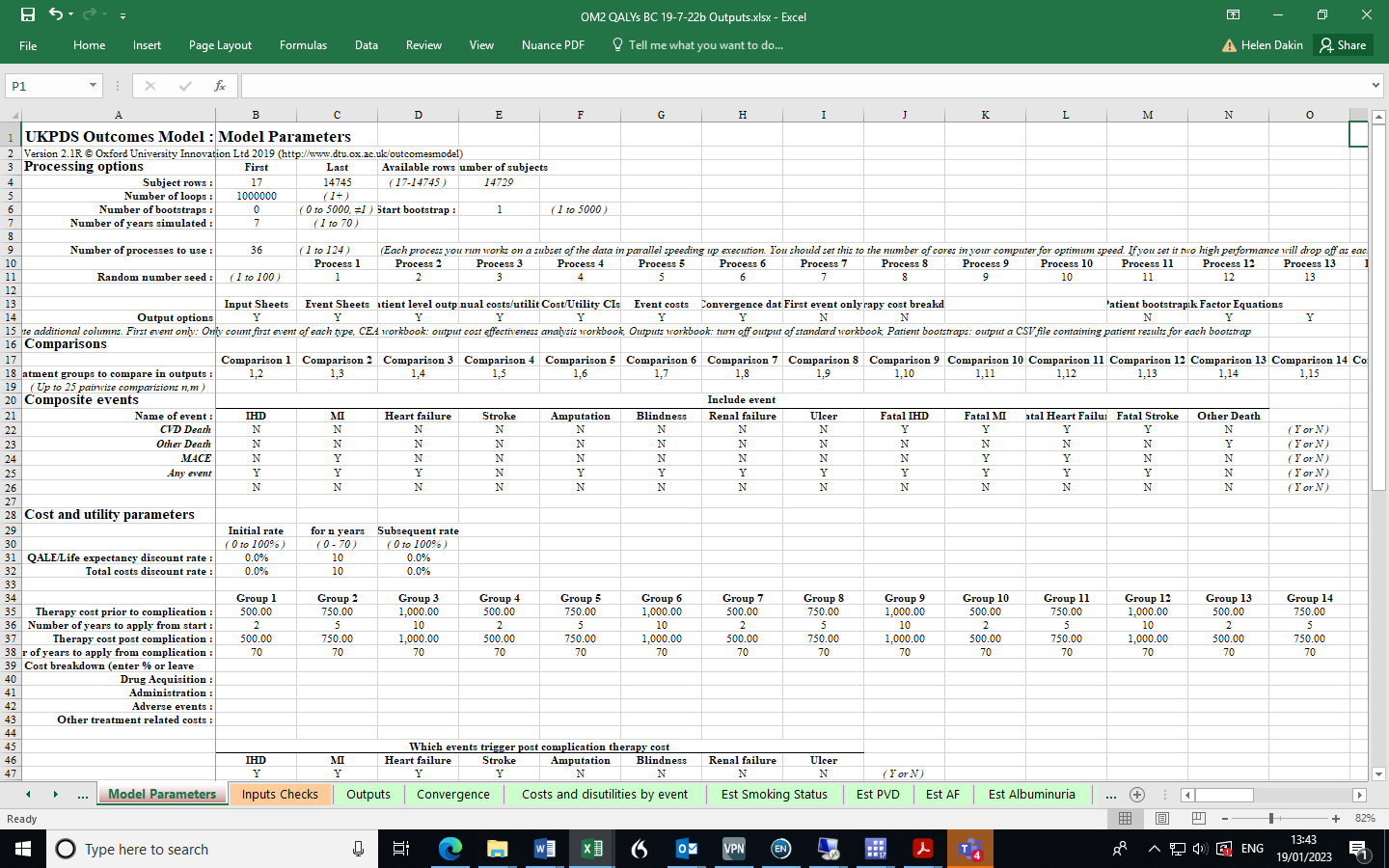


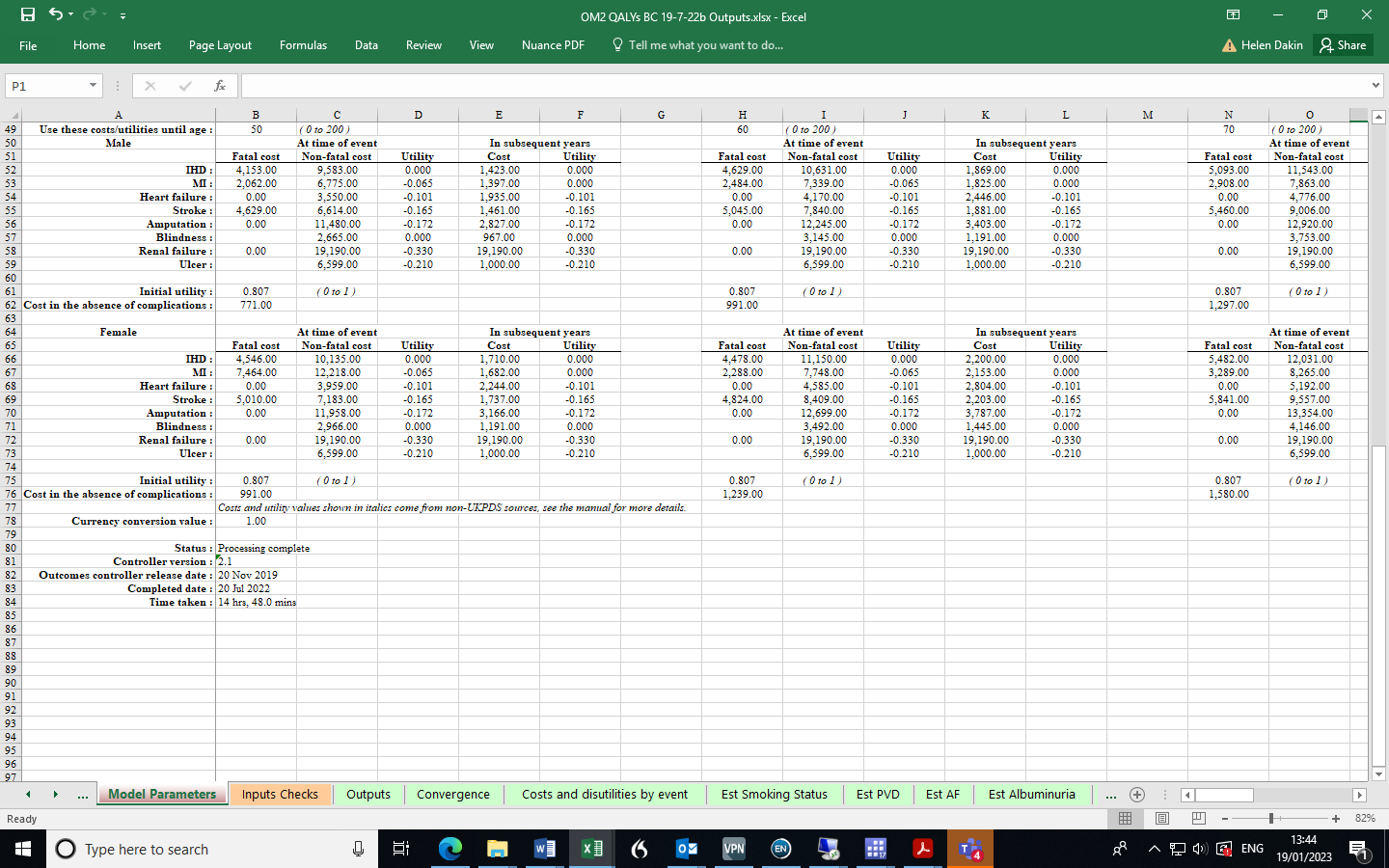


A separate run of the model was used to estimate the cumulative incidence of the first event of each type, which used the following options and set up a composite event “AnyEvent”, which included all events in the model and death from any cause.

**Figure S1.3:** Set up of UKPDS-OM2 ‘Model Parameters’ sheet for the run of the model that was used to estimate cumulative incidence of first events. The inputs used in our simulation are presented using the text and format of the UKPDS-OM2.1 input spreadsheet developed by Oxford University.^7^


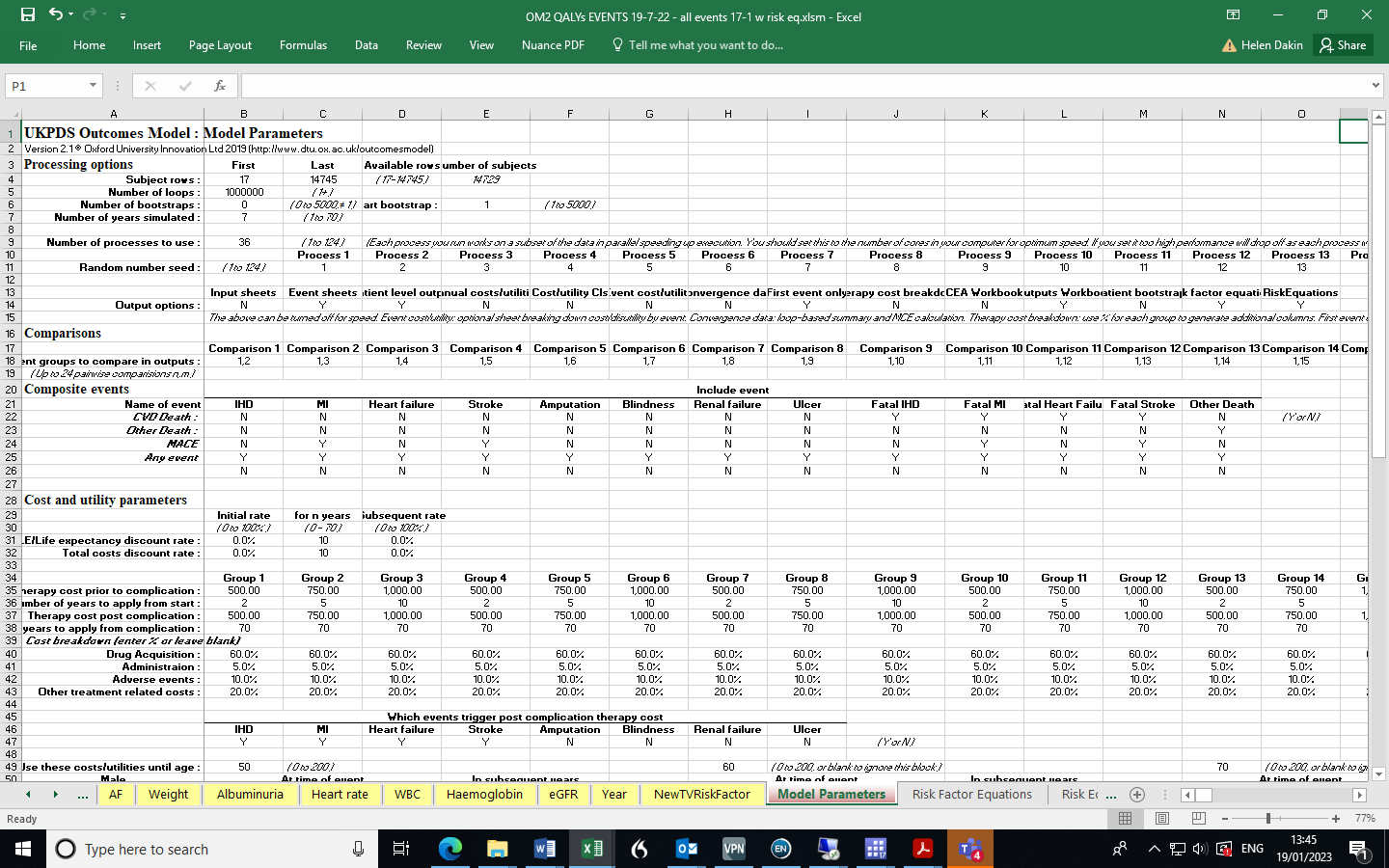


***Set up of UKPDS-OM 1.3 for simulations***

We used the standalone version of UKPDS-OM version 1.3 for the simulations. The set up for the base case simulation was:

**Figure S1.4:** Set up of UKPDS-OM version 1.3 for the base case analysis. The inputs used in our simulation are presented using the text and format of the UKPDS-OM1.3 graphical user interface developed by Oxford University.^11^ This figure is a screenshot of the UKPDS Outcomes Model user interface, version 1.3. Copyright Diabetes Trials Unit, University of Oxford 2010, reproduced with permission.


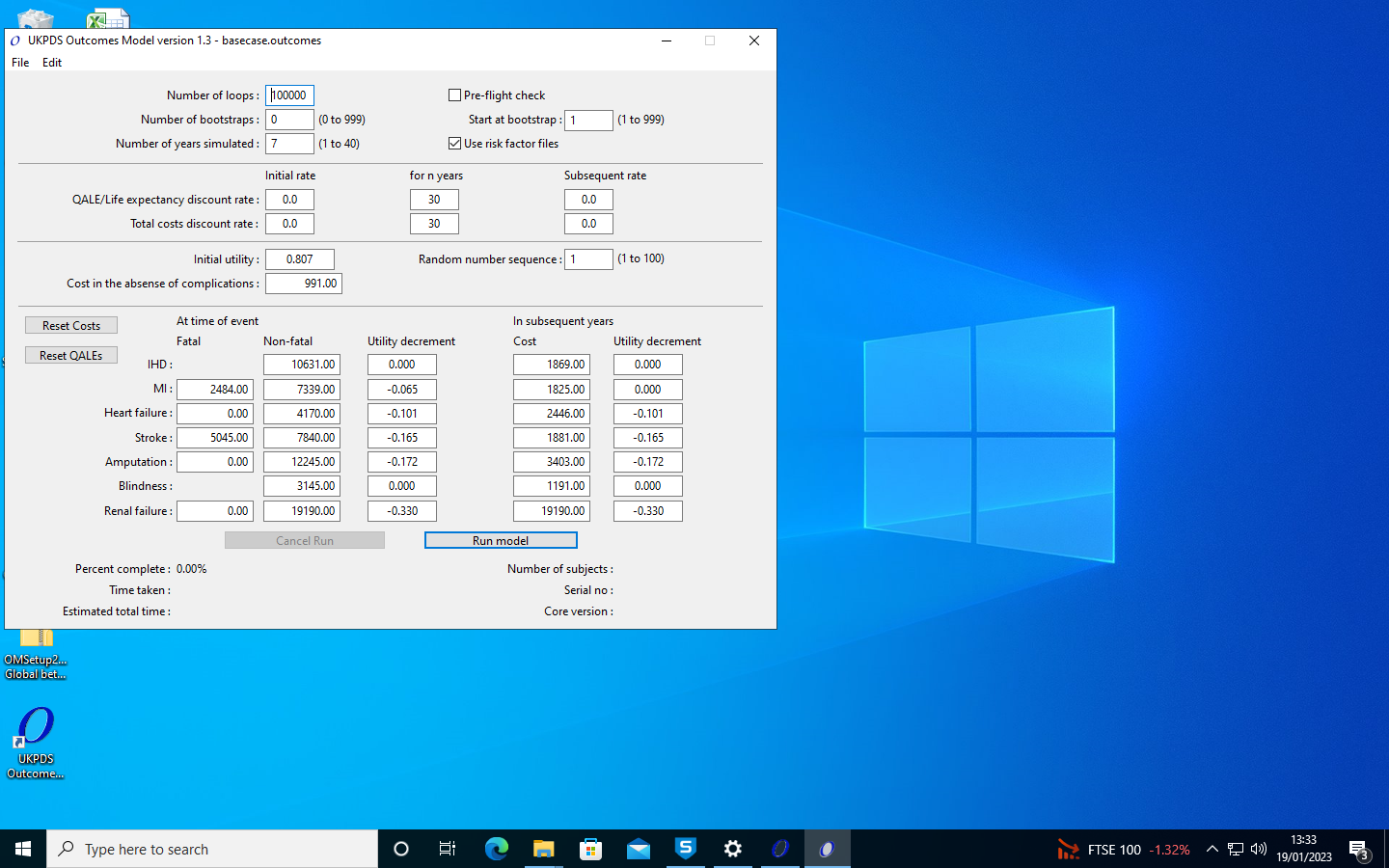


***Statistical software***

Stata version 17 (StataCorp, College Station, TX) was used for all analyses that were not done using UKPDS-OM1 or OM2.

***Estimation of cumulative incidence figures***

When estimating the model cumulative incidence, we took the annual incidence of each event predicted by UKPDS-OM for each patient and took the average of the annual incidence across all patients included in that analysis (e.g. averaging annual incidence of first MI in year 2 across all patients who had no history of MI at randomisation). We then summed the mean annual incidence over the years to date to get cumulative incidence.

***Baseline characteristics of EXSCEL sample***

Baseline characteristics of the EXSCEL sample have been described in detail previously.^4^

EXSCEL participants were randomised between 2010 and 2015 and followed until a final visit, which took place between December 2016 and April 2017 unless the participant had died, withdrew or was lost to follow-up earlier. 7% (1,041/14,729) of the sample died; 4% (552/14,729) withdrew or were loss to follow-up before the study ended and 89% (13,136/14,729) completed the study between December 2016 and April 2017.

Participants had a median age of 62.7 (interquartile range, IQR: 56.4, 68.8) years, median diabetes duration of 12 (IQR: 7, 18) years and were 38% female and 76% white ethnicity. Overall, 73% had at least one prior cardiovascular event (70% coronary artery disease, 24% peripheral arterial disease, 22% cerebrovascular disease) and 37% had no known cardiovascular disease.^4^

**Appendix 2: Code for estimating trial QALYs**

See separate file

**Appendix 3: Assumptions used to calculate QALYs within UKPDS-OM2.**

The base case analysis used the default utility inputs within UKPDS-OM2, since they were estimated on longitudinal data (UKPDS) using fixed-effects models that deal with omitted time-invariant variable bias^12^ (Table S3.1).

**Table S3.1:** Utilities and disutilities (utility reductions associated with clinical events) used in the analysis

|  | **Base case**^12^ | **Sensitivity Analysis 4**^13^ |
| --- | --- | --- |
| Initial utility | 0.807 | 0.785 |
| IHD* | -0.000 | -0.09 |
| MI: during year of event | -0.065 | -0.055 |
| MI: in subsequent years | -0.000 | -0.055 |
| Stroke* | -0.165 | -0.164 |
| (Congestive) heart failure* | -0.101 | -0.108 |
| Blindness (in one eye)* | -0.000 | -0.074 |
| (Diabetic foot) ulcer* | -0.210 | -0.170 |
| Amputation* | -0.172 | -0.280 |
| Renal failure* | -0.330 | -0.204 |

* The same disutility was applied during the year when the event took place and in all subsequent years.

The following assumptions are used within the UKPDS-OM2 and when estimating trial QALYs:

- Every participant is assumed to have the "initial utility" (0.807) when they enter the model (or at randomisation). Events that occurred >12 months before the start of the simulation are assumed to have no effect on initial utility.
- Events have additive effects on utility, with each event reducing utility by a fixed amount regardless of whether any other events have occurred.
- UKPDS-OM2 only predicts second events for MI, stroke and amputation. Within the model, participants who have a history of one or more MI at baseline can have up to 1 further MI during the model period, while participants who have no history of MI at baseline can have up to 2 MIs during the model period (and similarly for stroke and amputation). For any other event (ulcer, IHD, heart failure, renal failure, blindness), participants with no history of the event at randomisation can have up to 1 of each type of event during the simulation; participants with a history of ulcer, IHD, heart failure, renal failure or blindness are not predicted to have another event of that type during the model period. We mirrored this when calculating trial QALYs, by applying no additional disutility was applied to a third MI (or stroke/amputation), or a second ulcer (or blindness) when estimating trial QALYs.
- Two events of the same type give double the disutility. If a participant has 2 non-fatal strokes (or amputations), the model applies the disutility twice: for example, if a participant has a non-fatal stroke in Year 2 and in Year 3 (which has a utility decrement of -0.165 in the year of the event and in subsequent years), their utility after the second stroke was 0.477 (0.807 minus 0.165 minus 0.165). For MI, there is no disutility for subsequent years,^12^ so if a participant has non-fatal MI in Year 2 and Year 4 and no other events, they get the disutility of -0.065 in Year 2 (reducing their utility to 0.742 [0.807-0.065]) and then no disutility from MI in Year 3 (utility 0.807), then another disutility of -0.065 (reducing their utility to 0.742 [0.807-0.065]) when the second MI occurs in Year 4 and return to a utility of 0.807 from year 5.
- The disutilities of all events are applied from the beginning of that year, such that participants who have an MI in Year 1 and survived for the whole year experience 12 months with a utility equal to initial utility minus disutility for MI (0.807 minus 0.065=0.742 QALYs).
- Death will give half of the ongoing disutility from previous events. For example, participants who have an MI and die in Year 1 of a different event (e.g. fatal stroke) accrue QALYs for 6 months with a utility equal to initial utility minus disutility for MI (0.807 minus 0.065=utility of 0.742 QALYs for 6 months = 0.371 QALYs).
- No disutility is applied for the event that causes the participant’s death, because the interval between the event and death is assumed to be negligible. IHD, MI, heart failure, stroke, amputation and renal failure are assumed to be potentially fatal, whereas blindness and ulcer are assumed to not be potential causes of death. In the estimation of trial QALYs (and in UKPDS-OM2), if a participant experiences several potentially-fatal events during the final year of life, random numbers are generated to identify which of these events caused death.

***Methods for adjusting model QALYs for censoring and mortality***

The estimates of model QALYs in each year of follow-up already reflect the fact that in a proportion of loops, the participant will die during that year and therefore accrue QALYs for only half the year, regardless of whether they are censored. When calculating model QALYs in the year when the trial participant was administratively censored or withdrew, we therefore only reduced the QALYs for 1 minus the proportion of loops where the participant died in that cycle (${1-p}_{die}$). QALYs in the year when the participant was censored (${months}_{obsserved}$ into the year) equalled $UnadjustedQALYs\left( p_{die}+\left( 1-p_{die} \right)(\frac{{months}_{obsserved}}{12}) \right)$. For example, if a participant was censored at 3.4 years and the model predicts that they have a 20% chance of dying in Year 4, we would multiply the unadjusted Year 4 model QALYs by 20%+(1-20%)*0.4, so that the model prediction of QALYs for that year is reduced by 48% for the model loops where the participant survived Year 4, but not adjusted for the model loops where the participant died in Year 4 (because a half-cycle correction has already been made).

**Appendix 4: Sensitivity analysis methods**

Six sensitivity analyses tested the robustness of the analysis to changes in the methods (Table S4.1). UKPDS-OM1 predicts a maximum of one occurrence of each event type per participant per loop, while UKPDS-OM2 allows for second occurrence for three specific event types (MI, stroke and amputation). In the base case analysis, calculation of trial QALYs included the disutility of all events that can be predicted in UKPDS-OM2, including second events that cannot be predicted by UKPDS-OM1. For example, if a participant had two ulcers and three MIs during the trial period, disutilities were only applied to the first ulcer and the first two MIs, with no additional disutility being applied to subsequent events. For participants who have had one MI (or stroke/amputation) before randomisation, the UKPDS-OM2 will predict the probability of a second MI (or stroke/amputation); by contrast participants with a baseline history of heart failure are no longer considered to be at risk of heart failure.

**Table S4.1.** Summary of the methods for sensitivity analysis, number of Monte Carlo iterations (loops) and simulation time

|  | **Sample size** | **2^nd^ events for MI, stroke and amputation** | **Disutility associated with ulcer** | **Utility data source** | **QALYs in year of censoring** | **Disco-unting** | **Loops run (time taken) UKPDS-OM1** | **Loops run (time taken) UKPDS-OM2** |
| --- | --- | --- | --- | --- | --- | --- | --- | --- |
| **Base case** | 14,729 | If no prior history | Y | Alva et al^12^ | Y | 0% | 100,000 (6 hrs 3) | 1 million (14 hrs 50) |
| **Sensitivity analysis 1: including 2^nd^ events** | 14,729 | **Regardless of prior history** | Y | Alva et al^12^ | Y | 0% | 100,000 (6 hrs 3)* | 1 million (14 hrs)* |
| **Sensitivity analysis 2: excluding 2^nd^ events** | 8,269  (with no prior history of MI, stroke or amputation) | **None** | Y | Alva et al^12^ | Y | 0% | 100,000 (6 hrs 3)* | 100,000 (1 hr 41) |
| **Sensitivity analysis 3: excluding 2^nd^ events or ulcer** | 8,269  (with no prior history of MI, stroke or amputation) | **None** | **N** | Alva et al^12^ | Y | 0% | 100,000 (6 hrs 3)* | 100,000 (1 hr 39) |
| **Sensitivity analysis 4: Beaudet** | 14,729 | If no prior history | Y | **Beaudet et al**^13^ | Y | 0% | 50,000 (3 hrs 10) | 100,000 (1 hr 40) |
| **Sensitivity analysis 5: excluding QALYs in year censored** | 14,729 | If no prior history | Y | Alva et al^12^ | **N** | 0% | 100,000 (6 hrs 3)* | 1 million (14 hrs)* |
| **Sensitivity analysis 6: discounted** | 14,729 | If no prior history | Y | Alva et al^12^ | Y | **3.5%** | 50,000 (3 hrs 15) | 100,000 (1 hr 42) |
| **Sensitivity analysis 7: 1 year time horizon** | 14,729 | If no prior history | Y | Alva et al^12^ | Y | 0% | 100,000 (6 hrs 3)* | 1 million (14 hrs 50)* |
| **Sensitivity analysis 8: 3 year time horizon** | 14,729 | If no prior history | Y | Alva et al^12^ | Y | 0% | 100,000 (6 hrs 3)* | 1 million (14 hrs 50)* |
| **Sensitivity analysis 9: model life-years predicting trial QALYs** | 14,729 | If no prior history | Y | Alva et al^12^ | Y | 0% | 100,000 (6 hrs 3)* | 1 million (14 hrs 50)* |
| **Sensitivity analysis 10: model QALYs predicting trial life-years** | 14,729 | If no prior history | Y | Alva et al^12^ | Y | 0% | 100,000 (6 hrs 3)* | 1 million (14 hrs 50)* |

* This analysis used the base case run of the model

Sensitivity Analysis 1 included the disutilities associated with up to two MIs, strokes, amputations, blindness (in one eye) or ulcer and up to one heart failure or IHD diagnosis when estimating trial QALYs, regardless of patient history; it was hypothesised that this would worsen prediction accuracy for both models because it introduces a systematic difference in the methods for QALY calculation between trial and model QALYs.

Sensitivity Analysis 2 excluded disutilities from second MI, stroke or amputation in the estimation of both trial QALYs and model QALYs by setting the coefficients for second events to -1,000,000 (on a log-hazard scale) within our beta version of UKPDS-OM2 and focusing on the subset of participants with no prior MI, stroke or amputation. Sensitivity Analysis 3 extended Sensitivity Analysis 2 by also applying no disutility for ulcers in the estimation of both trial QALYs and model QALYs, since this event is not predicted in UKPDS-OM1. Sensitivity Analysis 4 used an alternative set of utilities from a systematic review.^13^ Sensitivity Analysis 5 excluded QALYs accrued in the year in which a participant was censored or withdrew from the study, but still included QALYs in years in which the participant died. Sensitivity analysis 6 discounted QALYs at 3.5% per annum. Sensitivity analyses 7 and 8 varied the time horizon, including only QALYs accrued in year 1 or in years 1-3 (for both model and trial QALYs).

Sensitivity analyses 9 and 10 used the base case model run and assumptions but were set up to be deliberately extremely biased to evaluate and compare the performance of different metrics of model performance. Sensitivity analysis 9 used model life years as a biased estimate of trial QALYs, while sensitivity analysis 10 used model QALYs as a biased estimate of trial life-years.

**Appendix 5: Additional results**

***Distributional plots***

Model QALYs from both UKPDS-OM1 and OM2 reflect the multi-peaked distribution of trial QALYs, although model predictions are smoother than those of the trial – probably due to averaging over many loops.

**Figure 5.1:** Histogram of trial QALYs, model QALYs for UKPDS-OM1 and model QALYs for UKPDS-OM2

**Figure 5.2:** Scattergraphs showing correlation between model and trial QALYs for (A) UKPDS-OM1 and (B) UKPDS-OM2. The appearance of vertical stripes in the graph arises from patients who died within the trial (since death is assumed to occur halfway through the year when estimating QALYs).

Convergence was assessed for the base case run of UKPDS-OM2 (Figure 5.3). Data on outcomes for each loop are not reported in UKPDS-OM1, so convergence could not be assessed, although there is no reason to expect UKPDS-OM1 to converge more slowly than UKPDS-OM2. This shows that the model results converge by around 50,000 loops – the minimum number run for any sensitivity analysis (Table S4.1). Running more than 50,000 loops for the base case analysis and UKPDS-OM2 sensitivity analyses provides an additional safeguard against Monte Carlo error but would not change the conclusions.

**Figure 5.3:** Convergence plot for 1 million loops in UKPDS-OM2.


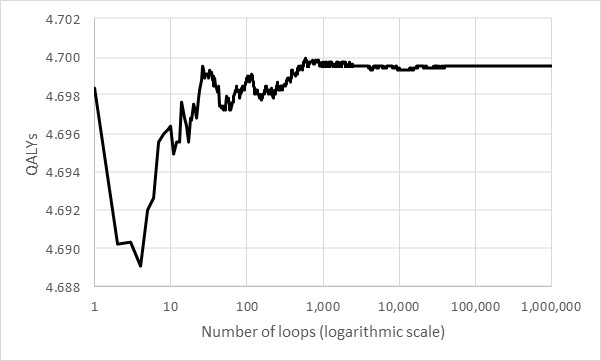


***Sensitivity analyses***

Allowing for second ulcers and blindness in the other eye when estimating trial QALYs (which are assumed to not arise within UKPDS-OM2) slightly lowered MSE for UKPDS-OM2 (Sensitivity Analysis 1; Table S5.2, Figure 2). However, prediction accuracy for UKPDS-OM1 was marginally worse in this analysis than in the base case. Sensitivity Analysis 2 excluded the impact of second MI, stroke and amputation, while Sensitivity Analysis 3 also excluded the impact of ulcer in order to more closely match the events predicted within UKPDS-OM1. Both models had higher Q^2^ within Sensitivity Analyses 2 and 3 compared with the base case (Figure 2): probably as the overall incidence of events was lower in this analysis (which focussed on patients with no history of MI, stroke or amputation). Using an alternative set of utility values from a systematic review^13^ (Sensitivity Analysis 4) reduced both model and trial QALYs by around 3% and slightly reduced MSE and Q^2^ for both models. Changing the methods used to account for censoring by excluding partially-observed years in which the participant was censored (Sensitivity Analysis 5) reduced model and trial QALYs by 14% by shortening the follow-up. In this analysis, MSE values were smaller and both Q^2^ and R^2^ were larger, although the improvements were greater for UKPDS-OM1. Discounting had negligible impact on Q^2^, but reduced MSE and MAE by a larger amount, due to the reduction in the standard deviation in trial QALYs. Reducing the time horizon to one or three years substantially reduced MSE, MAE and standard deviations around QALYs. However, Q^2^ was robust to this change, being slightly lower in the analyses with a shorter time horizon.

**Table S5.2:** Prediction accuracy: MSE for QALYs

|  | **No. pts** | **MSE for QALYs** | | **Mean (SD) QALYs** | | |
| --- | --- | --- | --- | --- | --- | --- |
|  |  | **UKPDS-OM1** | **UKPDS-OM2** | **Trial** | **UKPDS-OM1** | **UKPDS-OM2** |
| Base case scenario | 14,729 | 0.2526 | 0.2105 | 2.57 (1.09) | 2.42 (0.95) | 2.45 (0.95) |
| Trial QALYs including 2^nd^ MI, stroke, amputation, blindness and ulcer since randomisation regardless of patient history (sensitivity analysis 1) | 14,729 | 0.2535 | 0.2103 | 2.57 (1.09) | 2.42 (0.95) | 2.45 (0.95) |
| Excluding disutility from 2^nd^ MI, stroke or amputation (sensitivity analysis 2): pts with no prior MI, stroke or amputation | 8,269 | 0.1936 | 0.1544 | 2.70 (1.13) | 2.57 (1.02) | 2.61 (1.02) |
| Excluding ulcer and 2^nd^ events (not captured in OM1) (sensitivity analysis 3): pts with no prior MI, stroke or amputation | 8,269 | 0.1921 | 0.1516 | 2.70 (1.13) | 2.57 (1.02) | 2.61 (1.02) |
| Alternative utility values^15^ (sensitivity analysis 4)* | 14,729 | 0.2435 | 0.2044 | 2.49 (1.06) | 2.35 (0.92) | 2.37 (0.92) |
| Excluding QALYs in the year when patients were censored (sensitivity analysis 5) | 14,729 | 0.1745 | 0.1452 | 2.20 (1.09) | 2.08 (0.98) | 2.10 (0.98) |
| Discounting at 3.5% per annum (sensitivity analysis 6) | 14,729 | 0.2158731 | 0.179697 | 2.45 (0.99) | 2.31 (0.87) | 2.33 (0.86) |
| 1 year time horizon (sensitivity analysis 7) | 14,729 | 0.004 | 0.003 | 0.78 (0.10) | 0.77 (0.09) | 0.77 (0.09) |
| 3 year time horizon (sensitivity analysis 8) | 14,729 | 0.094 | 0.078 | 2.03 (0.53) | 1.94 (0.46) | 1.95 (0.45) |

Abbreviations: MSE, mean squared error; QALY, quality-adjusted life-year; SD, standard deviation.

SA1: Trial QALYs include second MI, stroke, amputation, blindness and ulcer since randomisation regardless of patient history.

SA2: Excluding disutility from second MI, stroke or amputation in UKPDS-OM2: 8,269 patients with no prior MI, stroke or amputation.

SA3: Excluding ulcer and second events from UKPDS-OM2 (which are not captured in OM1): 8,269 patients with no prior MI, stroke or amputation.

SA4: Alternative utility values for both UKPDS-OM1 and UKPDS-OM2: Initial utility, 0.785; IHD, -0.09; MI, -0.055; stroke, -0.164; heart failure, -0.108; blindness, -0.074; ulcer, -0.170; amputation, -0.280; renal failure, -0.204; disutility for subsequent years same as year of event.^13^

SA5: Excluding QALYs in the year when pts were censored for both trial and model QALYs.

SA6: discounting QALYs at 3.5% per annum.

SA7: 1 year time horizon.

SA8: 3 year time horizon.

**References for Appendices**

1. Hayes AJ, Leal J, Gray AM, et al. UKPDS outcomes model 2: a new version of a model to simulate lifetime health outcomes of patients with type 2 diabetes mellitus using data from the 30 year United Kingdom Prospective Diabetes Study: UKPDS 82. *55th Annual Meeting of the European Association for the Study of Diabetes, Barcelona* 2013; 56: 1925-1933. DOI: 10.1007/s00125-013-2940-y.

2. Holman RR, Bethel MA, Mentz RJ, et al. Effects of Once-Weekly Exenatide on Cardiovascular Outcomes in Type 2 Diabetes. *N Engl J Med* 2017; 377: 1228-1239. 2017/09/15. DOI: 10.1056/NEJMoa1612917.

3. Pagano E, Konings SRA, Di Cuonzo D, et al. Prediction of mortality and major cardiovascular complications in type 2 diabetes: External validation of UK Prospective Diabetes Study outcomes model version 2 in two European observational cohorts. *Diabetes Obes Metab* 2021; 23: 1084-1091. 2020/12/31. DOI: 10.1111/dom.14311.

4. Mentz RJ, Bethel MA, Gustavson S, et al. Baseline characteristics of patients enrolled in the Exenatide Study of Cardiovascular Event Lowering (EXSCEL). *Am Heart J* 2017; 187: 1-9. 2017/04/30. DOI: 10.1016/j.ahj.2017.02.005.

5. Gao N, Dakin H, Holman R, et al. Estimating Risk Factor Time Paths Among People With Type 2 Diabetes And QALY Gains From Risk Factor Management. *Pharmacoeconomics* 2024; (in press). DOI: 10.1007/s40273-024-01398-4.

6. Leal J, Alva M, Gregory V, et al. Estimating risk factor progression equations for the UKPDS Outcomes Model 2 (UKPDS 90). *Diabet Med* 2021: e14656. 2021/07/24. DOI: 10.1111/dme.14656.

7. University of Oxford Diabetes Trials Unit (DTU) and Health Economics Research Centre (HERC). UKPDS Outcomes Model User Manual: Version 2.2, <https://secure.dtu.ox.ac.uk/dl/?File=OM2.2Manual.pdf> (2023, accessed 8 December 2023).

8. Clarke PM, Gray AM, Briggs A, et al. A model to estimate the lifetime health outcomes of patients with type 2 diabetes: the United Kingdom Prospective Diabetes Study (UKPDS) Outcomes Model (UKPDS no. 68). *55th Annual Meeting of the European Association for the Study of Diabetes, Barcelona* 2004; 47: 1747-1759. DOI: 10.1007/s00125-004-1527-z.

9. Coleman RL, Gray AM, McGuire DK and Holman RR. Estimating cardiovascular risk and all-cause mortality in individuals with type 2 diabetes using the UKPDS Outcomes Model. *55th Annual Meeting of the European Association for the Study of Diabetes, Barcelona* 2019; 62: S152.

10. White IR, Royston P and Wood AM. Multiple imputation using chained equations: Issues and guidance for practice. *Stat Med* 2011; 30: 377-399. 2010/12/02. DOI: 10.1002/sim.4067.

11. University of Oxford Diabetes Trials Unit (DTU) and Health Economics Research Centre (HERC). UKPDS Outcomes Model User Manual: Version 1.3, <https://www.dtu.ox.ac.uk/outcomesmodel/UKPDSOutcomesManual.pdf> (2011, accessed 16 November 2021).

12. Alva M, Gray A, Mihaylova B and Clarke P. The effect of diabetes complications on health-related quality of life: the importance of longitudinal data to address patient heterogeneity. *Health Econ* 2014; 23: 487-500. DOI: 10.1002/hec.2930.

13. Beaudet A, Clegg J, Thuresson PO, et al. Review of utility values for economic modeling in type 2 diabetes. *Value Health* 2014; 17: 462-470. 2014/06/28. DOI: 10.1016/j.jval.2014.03.003.
